# Disruption of Kcc2-dependent inhibition of olfactory bulb output neurons suggests its importance in odour discrimination

**DOI:** 10.1038/ncomms12043

**Published:** 2016-07-08

**Authors:** Kathrin Gödde, Olivier Gschwend, Dmytro Puchkov, Carsten K. Pfeffer, Alan Carleton, Thomas J. Jentsch

**Affiliations:** 1 Leibniz-Institut für Molekulare Pharmakologie (FMP), Robert-Roessle Str. 10, 13125 Berlin, Germany; 2 Max-Delbrück-Centrum für Molekulare Medizin (MDC), Robert-Roessle Str. 10, 13125 Berlin, Germany; 3 Department of Basic Neurosciences, School of Medicine, University of Geneva, 1 rue Michel-Servet, 1211 Geneva 4, Switzerland; 4 NeuroCure Cluster of Excellence, Charité Universitätsmedizin, Charitéplatz 1, 10117 Berlin, Germany

## Abstract

Synaptic inhibition in the olfactory bulb (OB), the first relay station of olfactory information, is believed to be important for odour discrimination. We interfered with GABAergic inhibition of mitral and tufted cells (M/T cells), the principal neurons of the OB, by disrupting their potassium-chloride cotransporter 2 (Kcc2). Roughly, 70% of mice died around 3 weeks, but surviving mice appeared normal. In these mice, the resulting increase in the intracellular Cl^−^ concentration nearly abolished GABA-induced hyperpolarization of mitral cells (MCs) and unexpectedly increased the number of perisomatic synapses on MCs. *In vivo* analysis of odorant-induced OB electrical activity revealed increased M/T cell firing rate, altered phasing of action potentials in the breath cycle and disrupted separation of odour-induced M/T cell activity patterns. Mice also demonstrated a severely impaired ability to discriminate chemically similar odorants or odorant mixtures. Our work suggests that precisely tuned GABAergic inhibition onto M/T cells is crucial for M/T cell spike pattern separation needed to distinguish closely similar odours.

## Abstract (web summary)

Synaptic inhibition in the olfactory bulb (OB) is believed to play a role in odour processing. Here, the authors use a Pcdh21-driven Cre-line to disrupt KCC2 expression in OB mitral cells and find altered synaptic connectivity along with disrupted separation of odour-induced activity patterns.

## Graphical abstract

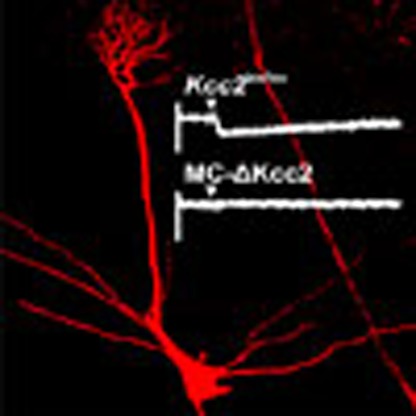

Discrimination between different, often very similar odorants does not only involve a very large diversity of olfactory receptors in the nose, but also requires precise neuronal processing in the olfactory bulb (OB). The OB is the first relay station of olfactory information in the central nervous system where odour-specific excitatory input from olfactory sensory neurons (OSNs) is received by glutamatergic mitral and tufted cells (M/T cells). These projection neurons relay the odour information to the olfactory cortices and other areas like the amygdala. M/T cells are extensively controlled by inhibitory input from three major subtypes of GABAergic interneurons, granule cells (GCs) and periglomerular cells^1^, as well as parvalbumin positive interneurons^2^,^3^. These interneurons form predominantly dendro-dendritic reciprocal as well as axo-dendritic synapses with M/T cells and mediate lateral and recurrent inhibition onto or between M/T cells^2^,^4^,^5^,^6^,^7^, which is important for correct processing and perception of odour information^8^,^9^,^10^.

Processing of olfactory information within the OB involves spatial and temporal coding^11^. Spatial coding is mediated by the distinct glomerular activity patterns elicited by odour-specific input from the OSNs to single glomeruli of the OB^12^,^13^,^14^. Temporal coding provides further levels of odour processing and discrimination, especially if the glomerular activity pattern for two odours is largely overlapping like with structurally similar odorants^15^,^16^,^17^. The temporal component involves synchronously spiking mitral cells (MCs), reflected by γ-frequency oscillations^10^,^18^,^19^, as well as separation of M/T cell activity pattern over time^20^,^21^,^22^. Both types of coding depend on GABAergic inhibition^4^,^10^,^21^,^23^. In particular, discrimination of similar odours or of odour mixtures seems to be influenced by the strength of inhibitory input from GCs, the predominant interneuron type in the OB. This conclusion was supported by experiments that changed GC activity with genetic, pharmacological or optogenetic tools^8^,^9^,^10^,^24^. For instance, changing the electrical excitability of GCs by disrupting specific glutamate receptor subunits with Cre-recombinase encoding viruses influenced the time needed for odour discrimination without affecting discrimination accuracy^8^.

GABA is the main inhibitory neurotransmitter in the mature central nervous system. It activates GABA_A_ receptor anion channels that, depending on the electrochemical Cl^−^ gradient, typically hyperpolarize the postsynaptic membrane or shunt excitatory currents. The appropriate low cytoplasmic Cl^−^ concentration is created by Kcc2 (potassium-chloride cotransporter 2), the main chloride extruder in mature neurons^25^,^26^,^27^. Knockdown or deletion of Kcc2 leads to an elevated intracellular chloride concentration ([Cl^−^]_i_) and decreases GABAergic driving force^25^,^26^,^27^. Depending on the brain region, Kcc2 may be expressed in rodents already at birth or is upregulated during the first postnatal weeks. In addition to its transport function, Kcc2 may also have morphogenic effects^28^,^29^,^30^,^31^ on spine morphogenesis^30^,^32^, synapse formation^33^,^34^ and proper localization of glutamatergic AMPA receptors^28^.

In the present study, we disrupted *Kcc2* within the murine OB specifically in M/T cells, the main projecting neurons. In contrast to other studies, which used viral transfection or injection of drugs into the OB, we targeted almost completely synaptic inhibition of M/T cells. This led to a reduced GABAergic hyperpolarization of MCs and, surprisingly, also to changes in synaptic connections at their somata. We show that these changes in the olfactory circuitry led to increased M/T cell firing rate and to deficits in M/T cell activity pattern separation *in vivo.* These changes were associated with a severely impaired ability of these mice to discriminate closely similar odours and odour mixtures.

## Results

### M/T cell deletion of potassium-chloride cotransporter 2

*Kcc2*^lox/lox^ mice, in which exons 2–5 are flanked by loxP sites^27^, were crossed with Pcdh21::Cre mice to obtain *Kcc2*^lox/lox^;Pcdh21::Cre mice. The Pcdh21 promotor was reported to drive Cre expression specifically in M/T cells of the OB^35^ and also in restricted parts of the cerebellum. We verified this expression pattern with R26R and Z/AP reporter mouse strains that express the *LacZ* gene and alkaline phosphatase, respectively, only in cells that produce the Cre recombinase. Although crosses with reporter mice revealed Pcdh21-driven Cre activity in the granular layer of the anterior part of the cerebellum (Supplementary Fig. 1a, b), immunolabelling for Kcc2 did not show a pronounced difference in Kcc2 protein expression between cerebella of control and *Kcc2*^lox/lox^;Pcdh21::Cre mice (Supplementary Fig. 1b). In contrast, specific deletion of Kcc2 in cerebellar GCs could be clearly visualized in a previous study^27^ that used Δα6::Cre mice for GC-specific *Kcc2* disruption. This comparison suggests that compared with Δα6-driven Cre expression, Pcdh21-driven Cre expression does not only occur in a smaller region of the cerebellum, but is also weaker in the anterior region where both promoters are active. As even Δα6-driven deletion of Kcc2 from virtually all cerebellar GCs resulted specifically in the impairment of consolidation of vestibulo-ocular learning without detectable effects on motor performance^27^, it is unlikely that a potential cerebellar deletion of Kcc2 will affect conclusions of the present study. As expected from the reporter stainings, Kcc2 protein expression appeared unchanged in the hippocampus and the piriform cortex between the genotypes (Supplementary Fig. 1c).

**Figure 1:**
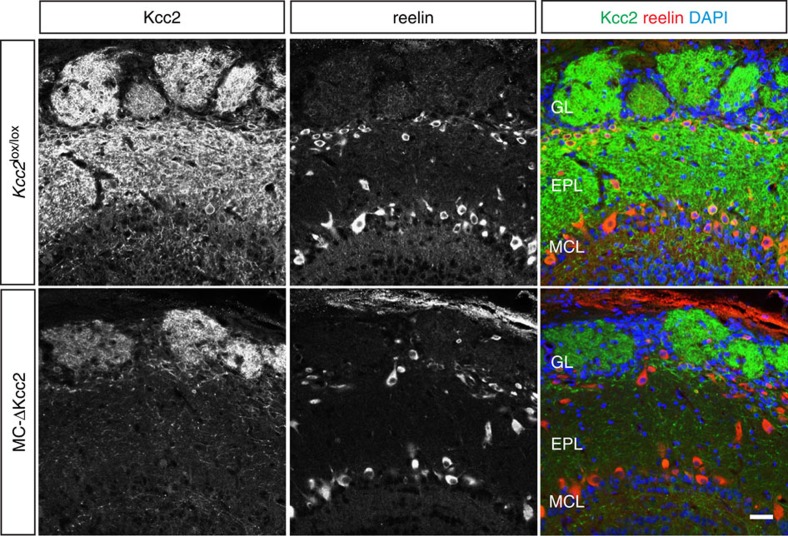
Cell-type specific deletion of *>Kcc2>* in mitral/tufted cells within the main OB. Immunofluorescent staining of Kcc2 (green) and mitral cell marker reelin (red) of coronal sections of the main OB from 5-week-old MC-ΔKcc2 mice compared with *Kcc2*^lox/lox^ littermates. Nuclei were stained with DAPI (blue). Scale bar, 30 μm. EPL, external plexiform layer; GL, glomerular layer; MCL, mitral cell layer.

Intense immunolabelling of Kcc2 in the external plexiform layer (EPL) and the glomerular layer (GL) of the OB indicated that Kcc2 is mainly localized to the lateral dendrites, dendritic tufts and to a lesser extent to the somata of MCs as seen by a lower signal intensity in the mitral cell layer (Fig. 1). A similar subcellular distribution with stronger Kcc2 expression in the dendritic compartment than at somata has been described previously in other neurons^36^,^37^. In *Kcc2*^lox/lox^;Pcdh21::Cre mice (subsequently called MC-ΔKcc2 mice), Kcc2 was almost completely absent from the EPL (Fig. 1). The remaining, less pronounced Kcc2 labelling in the MC-ΔKcc2 GL might originate from juxtaglomerular cells surrounding the glomeruli. Kcc2 mRNA can be detected in MCs as early as E15 (ref. 38). Kcc2 may be sufficiently active in lowering intracellular Cl^−^ concentration at P1 as GABA is able to inhibit action potential firing in MCs at that age^39^. The Pcdh21 promotor begins to drive Cre expression only postnatally, with robust expression from P14 onwards^40^. Accordingly, residual levels of the Kcc2 protein could be observed in the EPL of 1-week-old and 2-week-old MC-ΔKcc2 mice (Supplementary Fig. 2). In 5-week-old and older MC-ΔKcc2 mice, the EPL almost completely lacked Kcc2 expression compared with control mice (*Kcc2*^lox/lox^; Fig. 1 and Supplementary Fig. 2). About 70% of MC-ΔKcc2 mice died spontaneously at an age of around 3 weeks. However, mice surviving beyond that age had a normal life expectancy and displayed no obvious phenotype. The reason for this increased lethality could not be clarified but may be related to spurious Cre expression in those mice that die early. We used mice at P30 and older for all following experiments.

### Depolarizing shift of E_GABA_ in MCs after Kcc2 deletion

**Figure 2:**
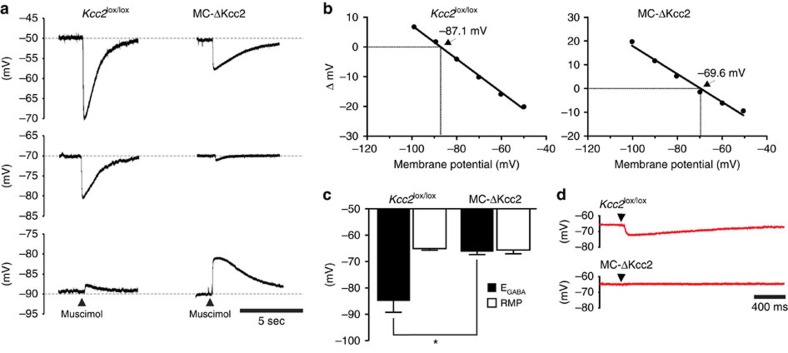
Effect of *Kcc2* disruption on E_GABA_ in mitral cells. (**a**) Example traces of voltage responses of MCs from *Kcc2*^lox/lox^ and MC-ΔKcc2 mice to muscimol measured in current-clamp mode. Cells were clamped to different resting membrane potentials by the injection of appropriate currents and muscimol was superfused by pressure application while keeping injected currents constant. (**b**) Linear regression analysis of muscimol-evoked responses (Δ mV) as a function of different initial holding potentials from experiments as in **a**. The holding potential at which muscimol evoked no voltage response indicates E_GABA_ (pointed out by arrows and numbers). (**c**) Averaged values of E_GABA_ and RMP obtained from these experiments (every single cell yields both values). E_GABA_ was significantly different between *Kcc2*^lox/lox^ and MC-ΔKcc2 MCs (*P⩽0.05, Mann–Whitney Test; *Kcc2*^lox/lox^: 8 cells and MC-ΔKcc2: 6 cells). RMP did not differ significantly between genotypes. (**d**) Example traces of MCs from *Kcc2*^lox/lox^ and MC-ΔKcc2 mice measured in current clamp mode with *I*=0 (that is, at resting membrane potential). E_GABA_ is below RMP values in *Kcc2*^lox/lox^ MCs, as indicated by hyperpolarizing response to muscimol, whereas almost no response is observed in MC-ΔKcc2 MCs (that is, E_GABA_≈RMP).

The effect of Kcc2 deletion on the GABA response of MCs was investigated by gramicidin-perforated patch clamp recordings that avoid equilibration of the intracellular ion concentrations with those in the pipette. Pressure application of the GABA_A_ receptor agonist muscimol onto MCs, the membrane potential of which was initially set to different voltages by appropriate injections of constant currents, revealed that the Cl^−^ reversal potential E_GABA_ was about 19 mV more positive in MCs lacking Kcc2 than in controls (*Kcc2*^lox/lox^: −84.9±4.3 mV, *n*=8 cells; MC-ΔKcc2: −65.6±1.4 mV, *n*=6 cells; Fig. 2a–c). A qualitatively similar, but smaller shift (−11 mV) was observed when E_GABA_ was calculated from voltage clamp experiments by applying muscimol at different voltage steps (*Kcc2*^lox/lox^: −80.2±2.15 mV, *n*=16 cells; MC-ΔKcc2: −69.7±0.84 mV, *n*=16 cells; Supplementary Fig. 3). The difference in the values for E_GABA_ obtained with these different methods might be explained by a slight inaccuracy of the offline access resistance correction. In MCs the bulk of Kcc2 is located to dendrites that display low electrical accessibility when cells are patch-clamped at their somata. Hence the shift in E_GABA_ might even be more pronounced in their dendritic compartments. When MCs were held at their resting membrane potential (RMP) in the current clamp mode (*I*=0), application of muscimol markedly hyperpolarized control MCs. Such a hyperpolarization was strongly reduced or absent in MCs of MC-ΔKcc2 mice (Fig. 2d). The RMP of MCs did not differ between genotypes (*Kcc2*^lox/lox^: −65.06±0.62 mV, *n*=8 cells; MC-ΔKcc2: −66.23±1.13 mV, *n*=6 cells; Fig. 2c), resulting in a reduced hyperpolarizing driving force for GABAergic currents in MCs lacking Kcc2. Nonetheless, GABA may still exert a net inhibitory effect in MC-ΔKcc2 MCs by electrically shunting depolarizing currents at the postsynaptic membrane.

### Increased number of GABAergic synapses at somata of MCs

**Figure 3:**
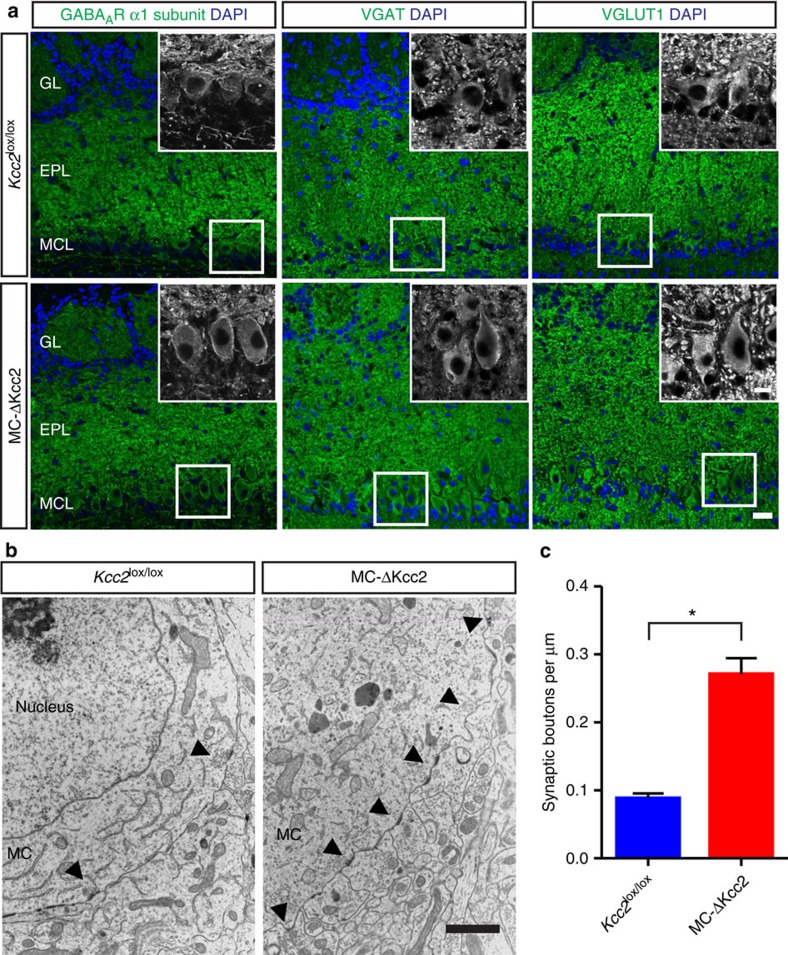
Increased number of synapses at mitral cell somata of MC-ΔKcc2 mice. (**a**) Immunofluorescent labelling of markers of inhibitory (GABA_A_ receptor subunit α1; vesicular GABA transporter, VGAT) and excitatory (vesicular glutamate transporter 1, VGLUT1) synapses in 10-μm-thick OB slices of MC-ΔKcc2 mice and *Kcc2*^lox/lox^ mice. Scale bar, 25 μm. Insets, higher magnification of areas indicated by white boxes. Scale bar, 10 μm. (**b**) Electron micrographs of perisomatic region of MCs from MC-ΔKcc2 mice and *Kcc2*^lox/lox^ mice. Arrows indicate contacting synaptic boutons (inhibitory and excitatory), characterized by a dense postsynaptic density and clustered vesicles either at the side of the MC or within the synaptic bouton. Scale bar, 500 nm. (**c**) Average number of synapses per membrane perimeter of MC-ΔKcc2 mice and *Kcc2*^lox/lox^ mice. *n*=4 mice per genotype (with 8–10 cells per mouse) were analysed. Mean+s.e.m. is displayed. Means are statistically different with *P⩽0.05, Mann–Whitney test. EPL, external plexiform layer; GL, glomerular layer; MCL, mitral cell layer.

The early gradual change of the GABA response from potentially excitatory to inhibitory may influence central nervous system development^41^. Consistent with the late expression of the Cre recombinase in MC-ΔKcc2 mice, their OBs lacked gross morphological abnormalities and displayed normal labelling for the M/T cell marker PGP9.5 (Supplementary Fig. 4). The coalescence of OSN axons to distinct glomeruli also appeared unchanged (Supplementary Fig. 5). The Kcc2 protein may have an ion transport-independent role in synapse formation^33^,^34^ and in the localization of postsynaptic receptors^28^. We, therefore, stained OB sections for markers of inhibitory and excitatory synapses. Surprisingly, labelling for the α1 GABA_A_ receptor subunit, which in the OB is only expressed in MCs and short axon cells^42^, was robustly increased in the perisomatic region of MCs of MC-ΔKcc2 mice, whereas in the EPL overall labelling intensity and pattern appeared unchanged (Fig. 3a). Likewise, labelling for vesicular transporters of GABA and glutamate (VGAT and VGLUT1) was enhanced around MC-ΔKcc2 MC somata (Fig. 3a). These findings suggested a larger number of perisomatic reciprocal synapses, which excite interneurons and inhibit MCs. This conclusion was confirmed by ultrastructural analysis that revealed an increased number of GC boutons contacting MC somata of MC-ΔKcc2 mice that could to a large part be characterized as reciprocal synapses (Fig. 3b,c and Supplementary Fig. 6a,b). In contrast synapse density was unchanged in the EPL between the genotypes (Supplementary Fig. 6c,d) and also the volume of the EPL was comparable between genotypes (*Kcc2*^lox/lox^: 0.125 mm^3^ versus MC-ΔKcc2: 0.129 mm^3^, determined using the method of Cavalieri with two mice per genotype).

**Figure 4:**
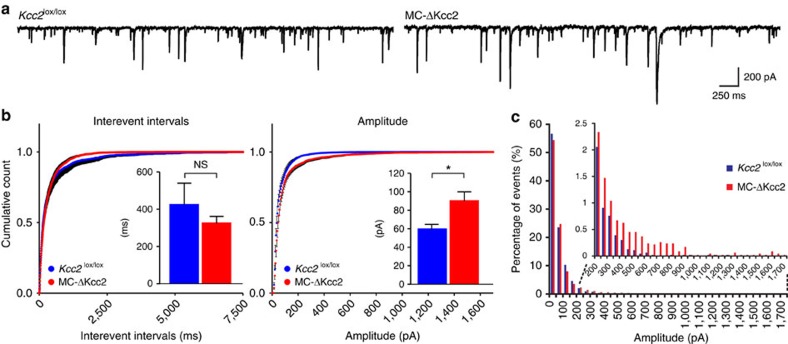
Effect of Kcc2 deletion on miniature inhibitory postsynaptic currents in MCs. (**a**) Sample traces of mIPSCs measured in MCs of MC-ΔKcc2 mice and *Kcc2*^lox/lox^ control mice, recorded in the voltage clamp configuration at −70 mV. (**b**) Mean cumulative probability and mean mIPSCs interevent intervals and amplitudes of MC-ΔKcc2 mice (*n*=20 cells) compared to *Kcc2*^lox/lox^ (*n*=18 cells). Mean+s.e.m. is shown. Mean amplitudes are statistically different between the genotypes (*P⩽0.05, Mann–Whitney test). Bin size for cumulative comparison was 5 pA and 5 ms, respectively. Cumulative probabilities are plotted with only positive or negative black error bars that represent s.e.m. (**c**) Frequency histogram showing percentage distribution of mIPSC amplitudes with a bin size of 50 pA. Inset displays histogram for amplitudes >200 pA. 4622 events were analysed per genotype. Distributions differ with an asymptotic significance (two-sided)<0.0005 (Kolmogorov–Smirnov test).

Consistent with the higher abundance of GABAergic synapses on MC-ΔKcc2 MC somata, the mean amplitudes of miniature inhibitory postsynaptic currents (mIPSCs) in these cells were significantly increased (Fig. 4) owing to a higher number of very large mIPSCs (>200 pA; Fig. 4c). These measurements were done in the whole-cell mode that abolishes the difference in [Cl^−^]_i_ between the genotypes. Interevent intervals, by contrast, showed no significant change (Fig. 4a,b). Spontaneous IPSC (sIPSC) amplitudes were increased as well (Supplementary Fig. 7a–c). The increased proportion of large-amplitude mIPSCs, together with unchanged mIPSC frequency, is consistent with a higher proportion of perisomatic inhibitory synapses that display better electrical accessibility when cells are patch-clamped at their somata. By contrast, there was no difference in either amplitudes or interevent intervals of excitatory postsynaptic currents between the genotypes (Supplementary Fig. 7d,e). This observation fits to the observed increase in reciprocal perisomatic synapses that excite GCs, which in turn inhibit MCs.

The deletion of Kcc2 from MCs seems to influence their inhibition in two different, apparently opposing ways: a decrease in GABA_A_ receptor-mediated hyperpolarization due to a shift in E_GABA_, and on the other hand an increased proportion of GABAergic synapses located at the soma of MCs that might potentially strengthen inhibition by more efficient electrical shunting close to the action potential initiation zone.

### Kcc2 deletion changed odour-evoked M/T cell responses

To test the effect of the deletion *in vivo*, we performed multiple single-unit recordings using tetrodes in awake, head-restrained mice^43^,^44^. We used structurally distinct odorants (that is, ethyl valerate, ethyl tiglate, octanal, hexanal) as well as binary mixtures of monomolecular odorants (that is, ethyl valerate/ethyl tiglate 60/40 and 40/60% relative ratios). We also used the enantiomers (+)-limonene: L+ and (−)-limonene: L− as well as compounds that differ in one methylene group, that is, octanol and heptanol.

**Figure 5:**
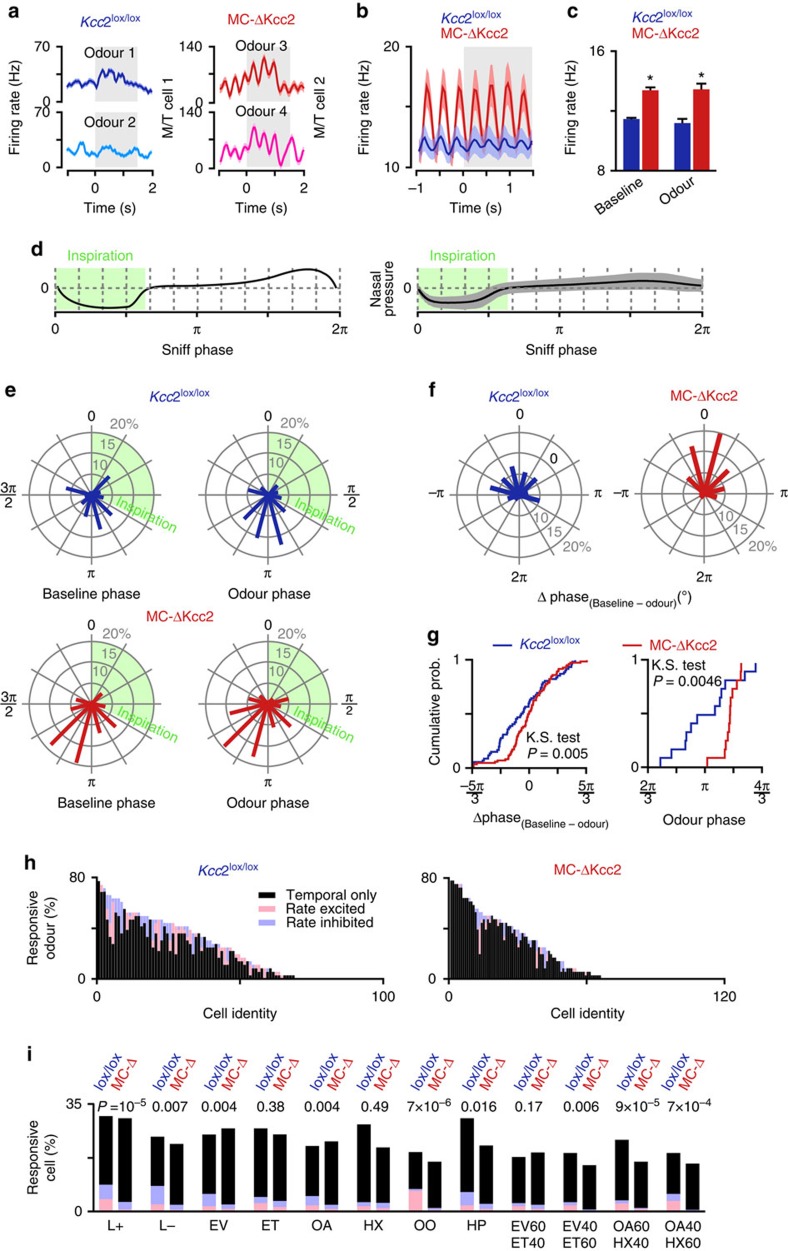
Kcc2 deletion from mitral/tufted cells alters response to odorants. (**a**) Representative peristimulus time histograms (PSTHs) showing responses to different odorants recorded in mitral/tufted (M/T) cells (PSTHs are mean±s.e.m., 10 trials). (**b**,**c**) Population firing rates averaged over the complete population of M/T cells (*n*=1,176 and 1,452 cell–odour pairs recorded from 7 *Kcc2*^lox/lox^ and 6 MC-ΔKcc2 mice, respectively). Baseline and odorant-evoked rates are significantly increased in MC-ΔKcc2 mice (Mann–Whitney test, *P⩽0.00005 and *P⩽0.0005 for baseline and odorant periods, respectively). Baseline firing rates are computed over three breaths pre-odorant application while odour histograms are computed for the first post-odour breath. (**d**) Traces of nasal airflow for a single breathing cycle (left) and the average breath across all animals (right). Inspiration is highlighted by the green box. (**e**) Circular plots of preferred phases of firing in the breathing cycle during baseline and odorant periods computed for M/T cells recorded in *Kcc2*^lox/lox^ and MC-ΔKcc2 mice. Only cell–odour pairs that presented a significant phasing were used (circular Rayleigh test, *P*⩽0.05). (**f**) Circular plots of change in preferred phasing between baseline and odour periods for all cells and odours. Only cell–odour pairs that presented a significant phasing were used. Delta phases are preferentially found around 0 in MC-ΔKcc2 mice indicating that phasing is not strongly changing when odorants are applied. (**g**) Left graph: Cumulative plot of delta phase between baseline and odour period for MC-ΔKcc2 and *Kcc2*^lox/lox^ mice (*n*=1,452 and 1,176 cell–odour pair, K.S. Kolmogorov–Smirnoff test). Right graph: Cumulative plot of preferred phase for all odours. (*n*=12 and 12 odours, Kolmogorov–Smirnov test). (**h**) Percentage of odorants evoking a significant response in at least one breath out of three after odour onset (*n*=98 and 121 cells for the *Kcc2*^lox/lox^ and MC-ΔKcc2 mice, respectively; see Methods section). (**i**) Percentage of cells displaying a significant response for each odour in at least one breath out of three after odour onset. Odorants evoked significantly less tonic responses in MC-ΔKcc2 mice (*χ*^2^-test).

MC-ΔKcc2 M/T cells displayed an increase in both baseline and odour-evoked firing rate (Fig. 5a–c). M/T cells change their phasing more often than their rate following odour application^43^,^45^. Thus, when the activity is averaged across all cell–odour pairs, phasic excitation and phasic inhibition compensate for each other which results in a net absence of global population firing rate change in comparison to the baseline. This effect was similarly observed for both genotypes (Fig. 5b). Breathing modulation of neuronal activity was also much more prominent in the MC-ΔKcc2 mice (Fig. 5b). An increased firing rate was observed when averaged over all tested odorants (Fig. 5c).

Interestingly, not only the rate was affected. The percentage of cell–odour pairs that were significantly phased to the breathing cycle in the baseline (30.7% odour–cell pairs) or in the odour period (37.2% odour–cell pair) in MC-ΔKcc2 mice was increased compared with *Kcc2*^lox/lox^ mice (20.2 and 12.1%, respectively, *χ*^2^-test, *P*=0.0013 and 0.0081; *n*=1,176 and 1,452 cell–odour pairs recorded from seven *Kcc2*^lox/lox^ and six MC-ΔKcc2 mice). M/T cells of MC-ΔKcc2 mice, hence, tended to be more active and phased to the sniff. While the preferred phase of *Kcc2*^lox/lox^ M/T cells drifted during the odour epoch compared with baseline, the preferred phase of M/T cells from MC-ΔKcc2 mice was much less affected by the odour stimulation (Fig. 5d–f). Hence, we observed a reduced odour-evoked change of phase by a distribution of delta phase (baseline–odour) skewed towards 0 degree for the negative values (Fig. 5f). In addition, the preferred phase for each odorant was significantly (Kolmogorov–Smirnoff test *P*=0.0046) less distributed over the sniff and tended to be drifted towards later phase (Fig. 5f,g). In other words, the Kcc2^lox/lox^ M/T cells spread their phase-related activity throughout the breathing period. In contrast, phase-related activity of M/T cells from MC-ΔKcc2 mice remained centred around the middle part of the sniff (180°), similarly to the baseline period (Fig. 5e,f). The percentage of odours that evoked a significant rate change for each MC was also reduced in the MC-ΔKcc2 mice (Fig. 5h). Finally, the percentage of excited and inhibited cells was also significantly reduced for nine odours (Fig. 5i).

As mice can adopt slow or fast sniffing, the preferred phase might possibly be modulated by this difference of breath regime. To test this possibility, we compared the duration of the three first sniffs in the odour epoch, and their respective pre-odorant period. MC-ΔKcc2 mice had more stereotyped breathing patterns since they were displaying less fast sniffing both during baseline and odour periods (Supplementary Fig. 8a–c). This difference might reflect an altered olfactory perception. It is important to note that the difference of breathing patterns cannot simply explain the differences of phasing observed between *Kcc2*^lox/lox^ and MC-ΔKcc2 mice (Fig. 5). Indeed no significant difference was observed when comparing the sniff-related preferred phase of cells in *Kcc2*^lox/lox^ mice for fast (<250 ms) and slow (>250 ms) sniffs (Supplementary Fig. 8d). Alternatively, the observed fast sniffing in *Kcc2*^lox/lox^ may be in part due to the novelty effect when a new odorant is presented to the mouse. Indeed, the odour-epoch sniffs of the first three trials are faster than the last six, in contrast to the MC-ΔKcc2 mice (Supplementary Fig. 8e). Despite that, cells recorded in *Kcc2*^lox/lox^ mice still do not display sniff phase preferences (Supplementary Fig. 8f). In summary, the differences of sniff duration cannot explain the phase changes observed between the two genotypes.

### *Kcc2* disruption impairs pattern separation between MCs

**Figure 6:**
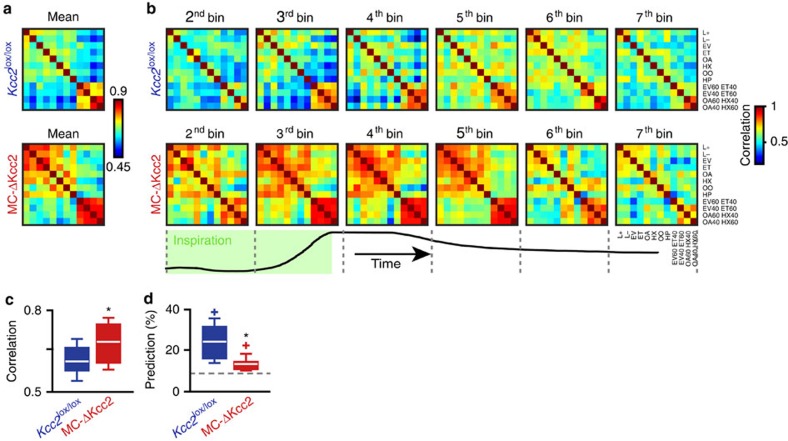
Increase in similarity of odorant-evoked ensemble responses by Kcc2 deletion in MCs. (**a**) Averaged correlation matrix of correlation matrices (from **b**) computed for 8 bins over the first breath for *Kcc2*^lox/lox^ (top matrices) and MC-ΔKcc2 (bottom matrices) mice. (**b**) Temporal evolution of the correlations for all possible pairs of mixtures during the first breath after odour onset for *Kcc2*^lox/lox^ (top matrices) and MC-ΔKcc2 (bottom matrices) mice. Correlation matrices were computed using vectors of firing rate averaged over consecutive 40 and 47 ms time windows for *Kcc2*^lox/lox^ and MC-ΔKcc2 mice, respectively. Only six time bins are shown for clarity. The breathing trace with the inspiration part is indicated below with the *y*-axis representing the nasal airflow. (**c**) Bar graph showing the average correlation computed over the first breath after odour onset for all odorant pairs for *Kcc2*^lox/lox^ and MC-ΔKcc2 mice (*n*=66 pairs, Mann–Whitney test, *P⩽0.00005). (**d**) Average prediction computed over the first breath after odour onset for all odorants pairs for *Kcc2*^lox/lox^ and MC-ΔKcc2 mice (χ^2^ test, *P⩽0.05). Dashed line corresponds to chance level (8.3% with the 12 odours tested). Data are presented as box plots (25th and 75th percentiles) showing the mean in white. Whiskers represent the 10th and 90th percentiles. Coloured plus signs represent maximum of prediction.

Works in zebrafish^20^ and in mouse^22^ suggest that pattern separation in the OB helps to disambiguate overlapping representations of activity evoked by similar odorants. Possibly, the reduced dynamic range of phase changes (Fig. 5e–g) increased the similarity between patterns of activity evoked by different odorants. We computed the activity rate of all single M/T cells in a population vector and calculated the Pearson's correlation between vectors of activity evoked by different odorants. All correlation values between pairs of odours were represented in a matrix computed over the first sniff after odour onset (Fig. 6a), or in matrices fractioned in the same number of sequential bins through the breathing cycle (Fig. 6b). The correlation between MCs in MC-ΔKcc2 mice was increased compared with control for most odour pairs tested (Fig. 6a,b). Accordingly, the overall correlation between odours, averaged over all tested odours, significantly increased (Fig. 6c). The possibility that the observed rise in correlation is simply a consequence of higher overall firing rate in MC-ΔKcc2 mice could be excluded (Supplementary Fig. 9).

In addition, odour responses of M/T cells in MC-ΔKcc2 mice were significantly less predicted by a simple classifier (Fig. 6d). In conclusion, *Kcc2* disruption reduced the diversity of M/T cell activity and interfered with the generation of diverse odour-evoked activity patterns.

### Kcc2 deletion impairs difficult odour discrimination

The ability of MC-ΔKcc2 mice to perceive and discriminate odours was tested in an associative olfactory learning task. Mice were trained in an automated olfactometer to distinguish two different odours, one of which was associated with a water reward that elicited licking responses^46^.

**Figure 7:**
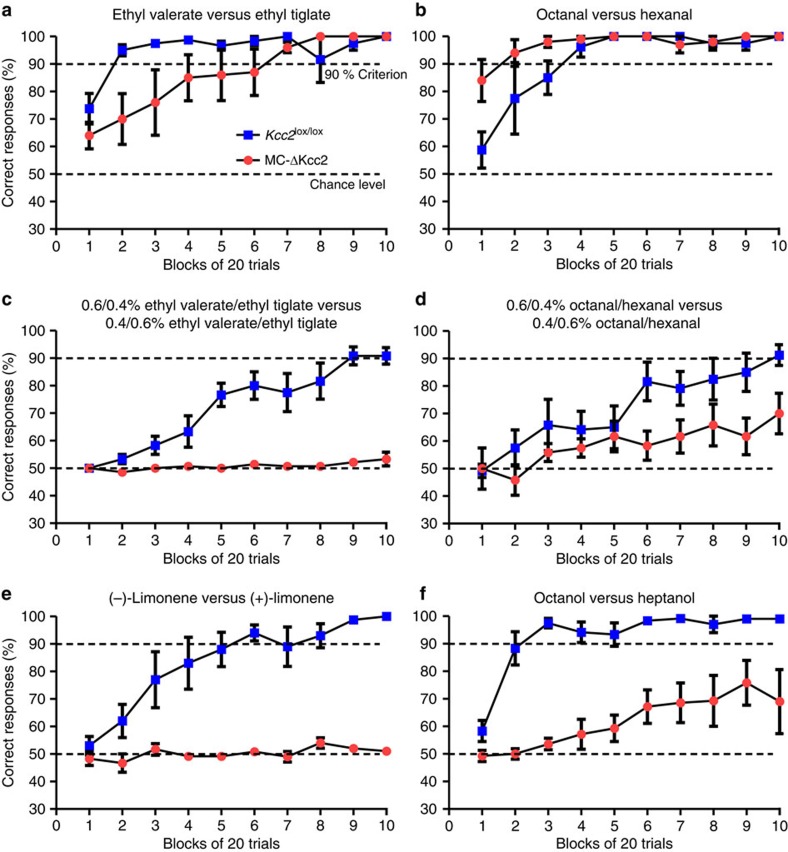
Impact of *Kcc2* disruption on odour discrimination. (**a**,**b**) MC-ΔKcc2 (*n*=4 mice) and *Kcc2*^lox/lox^ (*n*=5 mice) mice were able to discriminate between two structurally distinct odours, 1% ethyl valerate versus 1% ethyl tiglate (**a**) and 1% octanal versus 1% hexanal (**b**). Mice of both genotypes learned to discriminate these odours with an accuracy of more than 90% correct answers. (**c**,**d**) MC-ΔKcc2 mice show deficits in the ability to discriminate between mixtures of the odours tested in **a** and **b**. The following combinations were tested: 0.6/0.4 ethyl valerate/ethyl tiglate versus 0.4/0.6 ethyl valerate/ethyl tiglate (*n*=6 *Kcc2*^lox/lox^ against 7 MC-ΔKcc2 mice); 0.6/0.4 octanal/hexanal versus 0.4/0.6 octanal/hexanal. For both tasks MC-ΔKcc2 mice were not able to reach the 90% criterion in 10 tested blocks of 20 trials. (**e**) MC-ΔKcc2 mice show deficits in the ability to discriminate structurally similar odours. Mice were tested to discriminate enantiomers (−) limonene versus (+) limonene (1%). MC-ΔKcc2 (*n*=6 mice) were not able to perform better than chance level, while *Kcc2*^lox/lox^ mice (*n*=5 mice) reached 90% criterion after 6 blocks of 20 trials. (**f**) Mice tested to discriminate 1% octanol versus 1% heptanol. MC-ΔKcc2 mice (*n*=7) were not able to reach the 90% criterion in contrast to *Kcc2*^lox/lox^ mice (*n*=6 mice). Data are plotted as mean±s.e.m..

MC-ΔKcc2 mice were generally able to smell and to discriminate odours. In simple discrimination tasks with structurally distinct odorants (1% ethyl valerate versus 1% ethyl tiglate or 1% octanal versus 1% hexanal) both control and MC-ΔKcc2 mice were able to reach the criterion of 90% correct answers after a few blocks of 20 odour presentations (Fig. 7a,b). Control experiments using the diluent mineral oil in both odour channels excluded an influence of external cues (Supplementary Fig. 10).

More difficult tasks employed mixtures with different ratios of the previously tested odorants. Whereas control *Kcc2*^lox/lox^ mice learned to discriminate between 0.6/0.4% ethyl valerate/ethyl tiglate versus 0.4/0.6% ethyl valerate/ethyl tiglate or 0.6/0.4% octanal/hexanal versus 0.4/0.6% octanal/hexanal, and eventually reached the 90% criterion response in both cases, MC-ΔKcc2 mice did not perform better than chance level when tested for 0.6/0.4% ethyl valerate/ethyl tiglate versus 0.4/0.6% ethyl valerate/ethyl tiglate (Fig. 7c). Likewise, MC-ΔKcc2 mice did not reach the 90% criterion response when exposed to 0.6/0.4% octanal/hexanal versus 0.4/0.6% octanal/hexanal (Fig. 7d). Nor did MC-ΔKcc2 mice perform better than chance level in distinguishing the enantiomers L− from L+, whereas control mice reached the 90% criterion responses after only a few blocks of odour presentations (Fig. 7e). In contrast to *Kcc2*^lox/lox^ mice, MC-ΔKcc2 mice were also unable to reach the criterion response with 1% octanol versus 1% heptanol, compounds that only differ in one methylene group (Fig. 7f). Hence, deletion of Kcc2 did not abolish olfaction, but led to specific deficits in the discrimination of odorant mixtures and structurally similar odorants.

## Discussion

We investigated the impact of GABAergic inhibition of M/T cells, a crucial relay station in the OB, on the local circuitry involved in the processing of olfactory information. Modulation of M/T cells by inhibitory inputs is thought to refine the spatial representation of odours and thereby provide contrast enhancement of sensory information^4^. GABAergic inhibition in the OB also controls temporal coding of odour information by regulating the synchronization and activity pattern separation of simultaneously spiking M/T cells^20^,^23^. Accordingly, regulation of GABAergic input to M/T cells plays a role for odour discrimination in mice^8^,^9^,^10^.

Previous studies addressing the role of GABAergic inhibition in the OB with mouse models mainly approached the question by modulating the activity of GCs, the predominant GABAergic interneurons of the OB^8^,^9^,^10^, or by a combination of genetic manipulations using viral transfection and pharmacological manipulations, procedures that did not target all MCs or affected GABA_A_ receptors of other neurons as well^9^. In contrast, we globally decreased synaptic inhibition of the principal cells of the OB by increasing intracellular Cl^−^ concentration in virtually all MCs by deletion of Kcc2. Aside from decrease in GABAergic hyperpolarization, we found an effect on the subcellular distribution of GABAergic inputs with a higher number of reciprocal synapses contacting the perisomatic region of MCs. These changes in GABAergic inhibition led to an increased M/T cell firing rate and an impaired diversity of phased activity, and resulted in a lack of odour-evoked pattern separation. Unlike the more subtle phenotype of other mouse models targeting inhibition in the OB^8^,^9^,^10^, MC-ΔKcc2 mice showed severely impaired ability to distinguish structurally closely related odorants or different odorant mixtures. We believe that the global decrease of M/T cell inhibition in our mice importantly contributes to the stronger effect on olfactory learning tasks when compared with other mouse models^8^,^9^,^10^,^22^.

Rather than completely abolishing GABAergic synaptic inhibition by disruption of postsynaptic GABA_A_ receptors, we chose to reduce the strength of inhibition by disrupting the neuronal Cl^−^ extruder Kcc2 within the OB specifically in M/T cells. Kcc2 was almost completely deleted from M/T cells in mice older than P30. Since *Kcc2* disruption in cerebellar Purkinje and GCs elicits only a weak vestibulo-ocular learning phenotype^27^, we expect that the additional partial disruption in the cerebellum which we observed only in a small subset of GCs of MC-ΔKcc2 mice does not influence our results. Using sensitive reporter mice, we could not detect Pcdh21-driven Cre-recombinase activity in brain regions other than the OB and the cerebellum. Obviously, however, it is impossible to completely rule out the possibility that we missed spurious Cre-recombinase expression in a few neurons or small nuclei that might contribute to the observed high lethality of MC-ΔKcc2 mice at an earlier age. The fact that MC-ΔKcc2 mice performed indistinguishably from WT mice in relatively easy olfactory learning tasks aimed at distinguishing, for example, ethyl valerate from ethyl tiglate (Fig. 7a) or octanal from hexanal (Fig. 7b), demonstrated that these mice have no general defect in, for example, thirst, motor control and learning. Hence, any hypothetical influence in our mice from non-OB brain neurons should specifically affect the distinction between closely similar odours, or decisions based on such distinctions. In principle, such an influence appears possible. The OB receives input not only from olfactory epithelia, but also numerous centrifugal fibres from different brain areas. These include feedback loops involving olfactory cortex neurons (from the anterior olfactory nucleus and the piriform cortex), which directly excite OB GCs, or result in feedforward inhibition of GCs through interposed short axon cells, or can, to some extent, also excite MCs^47^,^48^. There is also input from neurons in the basal forebrain (horizontal limb of the diagonal band of Broca (HDB) and magnocellular preoptic area (MCPO)), which includes both cholinergic and GABAergic fibres^24^. Nunez-Parra *et al*.^24^ have also selectively silenced GABAergic neurons of the HDB/MCPO with inhibitory chemogenetic tools and observed a selective deficit in discrimination of closely related odours. Speculating further, there might be, for example, a change in neuronal circuits setting a threshold for the decision to lick. However, as we did not observe Cre expression in brain areas of MC-ΔKcc2 mice other than the OB and restricted parts of the cerebellum, and since similar, but weaker effects were observed by others when interfering in different, and often less complete ways with M/T cell inhibition, the most straightforward interpretation of our data is that the inability to distinguish closely similar odours results solely, or very predominantly, from reduced inhibition of M/T cells.

As observed in other neurons^25^,^27^, deletion of Kcc2 significantly increased [Cl^−^]_i_ in MCs. Whereas the pronounced reduction of the hyperpolarizing GABA response indicated a severely reduced synaptic inhibition of MCs, GABA-mediated inhibition may be partially preserved by a weaker shunting effect^49^,^50^,^51^.

The shunting inhibition of MC-ΔKcc2 MCs may be enhanced by the increased number of inhibitory synapses on their somata where they more efficiently modulate MC excitability than at dendritic sites^52^,^53^. Furthermore, the increase in somatic GABA receptors may influence somatic chloride concentration and hence the driving force for GABAergic currents during sufficiently high neuronal activity or in the presence of tonic GABA^54^.

The unchanged interevent intervals of mIPSCs in MC-ΔKcc2 MCs argue against a significant overall increase in synapse number. Compared with the large number of synapses on lateral dendrites, the increased number of perisomatic synapses appears small. However, the larger proportion of somatic synapses, which are electrically better accessible in our patch-clamp experiments, may explain the increased proportion of large-amplitude mIPSCs in MC-ΔKcc2 MCs.

Several studies suggested an ion transport-independent role of Kcc2 on spine maturation^30^,^32^, synapse formation^33^,^34^ and receptor localization in synaptic spines^28^. This effect, which is probably mediated by Kcc2 binding to the cytoskeleton-associated protein 4.1 (refs 28, 29, 30), is not seen universally as no changes in synapse density or morphology were found on *Kcc2* deletion in cerebellar Purkinje and GCs^27^. We do not know whether a loss of Kcc2/cytoskeleton interactions contributes to the observed shift of synapses to the perisomatic region of MC-ΔKcc2 MCs. This shift may represent a homoeostatic mechanism that partially compensates for the decreased inhibition of MCs. Compared with other brain regions, OB circuits may adapt more easily because of a constant stream of neuronal progenitors from the subventricular zone to the OB where these cells differentiate to interneurons and replace existing ones throughout life^55^. Likewise, in a mechanism called synaptic scaling, neurons can maintain a specific level of network activity by adjusting the density and strength of individual synapses^56^,^57^. Interestingly, the density of perisomatic synapses on mitral cells was previously found to be reduced on sensory deprivation^58^, suggesting a change in the density of perisomatic synapses as a more general mechanism in the regulation of MC excitability.

In accordance with reduced GABAergic inhibition, MC-ΔKcc2 M/T cells displayed an overall increased firing rate, either during baseline or odour-evoked activity (for the entire odour set tested). This suggests that the larger number of perisomatic synapses, which are expected to be inhibitory mostly by shunting^59^, cannot compensate for the reduction or loss of hyperpolarizing GABAergic currents. Population rate activity was unchanged between baseline and odour presentation in both genotypes as reported previously for awake mice^43^,^45^. More interestingly, MC-ΔKcc2 M/T cells displayed a more phased activity compared with controls. Despite this overall increased phased activity, and although MC-ΔKcc2 M/T cells were able to change their spike timing distribution through the sniff period (Fig. 5h), the preferred phase remained much more similar before and during the course of odour presentation. This reduced the diversity of the M/T cell response of MC-ΔKcc2 mice.

Hence, synaptic inhibition of M/T cells is essential to maintain a multiplexed coding strategy based on rate and phase shift^60^,^61^. The impaired inhibition resulted in a poor diversity of neuronal activity and led to a depleted odour-specific pattern generation as shown by the increased overall correlation between odorants. The representation of each odorant in the odour coding space resembled each other, leading to an increased mismatch of prediction when using a simple classification algorithm. These results emphasize the importance of an optimal pattern separation^20^ and more importantly, the role of GABAergic inhibition for sculpting odour-evoked patterns of activity, as previously suggested *in vitro*^21^,^62^,^63^ and *in vivo*^22^.

MC-ΔKcc2 mice could still smell and reliably distinguish structurally dissimilar odorants, but had severe difficulties in discriminating chemically similar odours or odour mixtures. By contrast, mice whose GC excitability was changed by virus-mediated excision of specific glutamate receptor subunits, could still discriminate between similar odours^8^. However, an increase or decrease of interneuron excitability correlated with an increased or decreased speed, respectively, of odour discrimination. In another study^9^ injection of low doses of the GABA_A_ receptor antagonist picrotoxin into the OB decreased, but did not abolish the ability of mice to discriminate enantiomers. However, this pharmacological manipulation does not completely target MCs. Global deletion of the β3-subunit of the GABA_A_ receptor, which specifically disrupts GABA_A_ receptors in OB GCs because these cells lack other β-subunits, entailed complex changes in olfactory experiments^10^. While all three mouse studies suggest a role of GABAergic inhibition of M/T cells in the discrimination of similar odours, our mice, in which we have globally targeted the inhibition of M/T cells, display the strongest effect on odour discrimination.

Temporal coding involves synchronization of simultaneously active M/T cells on one hand and pattern decorrelation of M/T cells over time on the other hand. Pattern separation is involved in the processing and discrimination of similar odours^22^,^63^,^64^. GABAergic inhibition plays a fundamental role in this process as shown in studies from zebrafish and in experiments in acute OB slices of mice^20^,^21^,^63^. As recently described^22^, our results confirm that a proper pattern separation helps mice to discriminate between odorants under normal conditions (*Kcc2*^lox/lox^ mice). For some odour pairs, the reduced pattern decorrelation does not predict a reduction of discrimination learning in the MC-ΔKcc2 mice. For instance, EV60/ET40 and ET60/EV40 are not discriminated by MC-ΔKcc2 mice while their amount of pattern correlation is not the highest. One reason might be our small-cell sampling. Indeed, it may not reflect the complete cell population recruited to encode a particular odorant. Another reason might be related to small differences in odour composition due to differences in olfactometers used during recording and behavioural experiments. Despite that, our results support the idea that the generation of a proper temporal activity pattern is crucial to build a correct olfactory representation of similar odours.

Recent experimental work and models reported that GL GABAergic interneurons modulate mitral cells phasing over the sniff (theta oscillation)^65^,^66^, while few or no effect of GCs has been observed on such slow oscillation but rather on gamma synchrony^66^. However, these observations are mainly based on anaesthetised preparations, in which M/T cells activity switches from a natural phasic temporal regime to a more artificial tonic rate regime^43^,^45^,^67^. Moreover, distribution of GC phasic activity across the sniff time course is also strongly depleted^68^, which probably does not reflect a physiological firing regime. In fact, GCs in awake mice display a much broader odour-evoked tuning likely helping to improve the diversity of MC responses over the sniff time course. This has been confirmed by recent work showing that GCs play a crucial role in enhancing M/T cells diversity of response and further driving similar odorant discrimination^22^.

In summary, our work suggests that proper odour discrimination needs correctly tuned GABAergic inputs onto M/T cells. Decrease of GABAergic hyperpolarization, as observed in MCs of MC-ΔKcc2 mice, was associated with a strong impairment of discrimination between related odour pairs or odour mixtures, an effect that is very likely due to the observed deficits in pattern generation. Somatic localization or strengthening of GABAergic synapses in mitral cells of MC-ΔKcc2 mice might partially compensate for the decrease in the driving force of the GABAergic currents by increased shunting inhibition at the soma. Nevertheless, this does not rescue the impairment in M/T cell pattern separation and odour discrimination.

## Methods

### Mice

All animal experiments were approved by LAGeSo, Berlin, Germany, or Swiss Federal Act on Animal Protection and Swiss Animal Protection Ordinance. *In vivo* experiments were approved by the University of Geneva and Geneva state ethics committees (authorizations 1007/3387/2 and GE/156/14).

*Kcc2*^lox/lox^ mice^27^ were crossed to Pcdh21::Cre mice (donated by Nathaniel Heintz via MMRRC; strain: Tg(Cdhr1-cre)KG66Gsat/Mmucd) to obtain M/T cell-specific deletion of Kcc2. Mice were kept in a mixed genetic background. MC-ΔKcc2 showed a high lethality of about 70% at an age of about 3 weeks for unknown reasons. Surviving mice had normal life expectancy and were phenotypically undistinguishable from *Kcc2*^lox/lox^ control mice and showed no signs of malnutrition. MC-ΔKcc2 mice were compared with *Kcc2*^lox/lox^ littermates as controls. Age of the animals used for experiments was P30 and older. For verification of Cre expression pattern, Pcdh21::Cre mice were crossed to Rosa26R Cre reporter mice (B6.129-Rosa26^tm^, obtained from Fred Hutchinson Research Center) and Z/AP reporter mice^69^.

### Immunohistochemistry and histology

Mice were anaesthetised and perfused with PBS, followed by 4% paraformaldehyde in PBS. Brains were postfixed in 4% PFA for 45 min and transferred to 30% sucrose for 24–48 h. Coronal and horizontal slices (10 μm) of frozen tissue were prepared with a Cryostat (Microm, HM560), blocked with 5% normal goat serum in 0.1 M phosphate buffer with 0.25% Triton-X100 for 2 h at room temperature, and then incubated with primary antibodies overnight at 4 °C. The following primary antibodies were used: rabbit anti-Kcc2 (1:500, against a C-terminal peptide: KNEREREIQSITDESC^29^), mouse anti-reelin (G10, 1:1,000) (a gift from Andre Goffinet, Université de Louvain), mouse anti-PGP9.5 (1:100, Abcam, ab8189), mouse anti-VGAT (1:250, Synaptic Systems, Cat.-No. 131011), guinea pig anti-VGLUT1 (1:250, Synaptic Systems, Cat.-No. 135304) and rabbit anti GABA_A_ receptor α1 subunit (1:250, Millipore, Cat.-No. 06-868). Secondary antibodies were coupled with Alexa-488 or Alexa-555 (1:1,000, Molecular Probes). DAPI (Invitrogen) was used for nuclear staining. Incubation time of secondary antibodies was 4 h at room temperature.

Determination of OSN coalescence and staining of reporter mice are described in detail in Supplementary Information.

### Electron microscopy

MC-ΔKcc2 mice and *Kcc2*^lox/lox^ littermates were anaesthetised and perfused transcardially with 4% formaldehyde and 2.5% glutaraldehyde in 0.1 M PB. Brains were isolated and postfixed in the same solution overnight at 4 °C. After rinsing in PBS, brains were embedded in 5% agar and OBs were sliced coronally (200 μm thick) with a vibratome. Slices were post-fixed in 1% OsO_4_ and 1.5% K-hexacyanoferrat, dehydrated in a methanol gradient and propylene oxide, and flat embedded in Epoxy resin. After polymerization the mitral cell layer or EPL were localized on semithin sections, trimmed and ultrathin sectioned. Sections were collected on mesh grids and analysed using a Zeiss 900 transmission electron microscope. Mitral cells were identified by their prominent size and presence of reciprocal synapses. Mitral cells sectioned at the nuclear level were photographed at × 12,000 magnification with Morada G2 digital camera. The number of reciprocal perisomatic synapses was normalized to the somata perimeter. 8–10 cells per animal and 4 animals per genotype were analysed. Synapses per area (μm^2^) were quantified in the neuropil of the EPL. On average 500 μm^2^ of neuropil per animal were analysed.

### Slice electrophysiology

Horizontal slices (250 μm) of the OB were prepared from mice older than P30. Brains were quickly removed from decapitated mice and cut with a vibratome (Leica) while being continuously submerged in ice-cold, carbogen-gassed, sucrose-based artificial cerebrospinal fluid (ACSF) containing (in mM): 83 NaCl, 2.5 KCl, 26.5 NaHCO_3_, 1 NaH_2_PO_4_, 3.3 MgCl_2_, 22 glucose, 72 sucrose and 0.5 CaCl_2_. Slices were then transferred to normal ACSF at 35 °C containing (in mM): 125 NaCl, 2.5 KCl, 25 NaHCO_3_ 25, 1.25 NaH_2_PO_4_, 1 MgCl_2_, 11 Glucose and 2 CaCl_2_ for 30 min and equilibrated to room temperature until transfer to the recording chamber. MCs were identified by their location and morphology with the use of differential interference contrast (DIC) video microscopy.

RMP and E_GABA_ measurements were done by gramicidin-perforated patch-clamping. Pipette solution contained 150 mM KCl and 10 mM Hepes (pH 7.3 with KOH). Gramicidin was added to a final concentration of 12–50 μg ml^−1^. Pipette resistance was 2–6 MΩ. Recordings with an access resistance >50 MΩ were excluded from analysis. For measurements of E_GABA_ and RMP, 1 μM tetrodotoxin (Tocris) was added to the ACSF. Membrane potentials were recorded with a Multiclamp 700B amplifier (Molecular Devices) in voltage clamp or current clamp mode.

RMPs were measured in the current clamp mode without any current injection (*I*=0 pA).

The reversal potential of GABAergic currents (E_GABA_) was measured in current clamp mode similar to previously described^70^. Membrane potential was set to voltages from −100 mV to −50 mV in 10 mV steps by current injections. At each membrane potential muscimol (Tocris) was applied focally by pressure application (50 ms, 4–6 psi, Pressure System IIe, Toohey Company) from a pipette (2–4 MΩ) with the tip located close to the soma. MCs were kept at *I*=0 pA for 45 s between current injections to allow washout of muscimol and equilibration of Cl^−^ levels. E_GABA_ was determined as the membrane potential at which muscimol-evoked voltages were 0 mV. Alternatively, E_GABA_ was measured in voltage clamp mode (Supplementary Fig. 3). MCs were clamped to voltages from −130 mV to −40 mV in steps of 10 mV with a step duration of 10 s. At each voltage step 50 μM muscimol (Sigma) was applied focally by pressure application from a pipette (2–4 MΩ) with the tip located close to the soma. Between voltage steps cells where clamped at −70 mV for 35 s to allow washout of muscimol and equilibration of Cl^−^ levels. Potentials were corrected offline for access resistance. Access resistance varied between 15–46 MΩ and was checked for stability directly before and after the measurement. Membrane resistance was not significantly different (*P*=0.153, Mann–Whitney Test) between *Kcc2*^lox/lox^ (Rm=55.1±9.4 MΩ) and MC-ΔKcc2 (Rm=38.5±4.8 MΩ) mice and values were stable before and after the measurement.

mIPSCs and sIPSCs were measured in the whole-cell configuration. MCs were clamped at −70 mV. ASCF was supplemented with 1 μM tetrodotoxin (Tocris), 10 μM NBQX (Enzo Life Science), 50 μM D-AP5 (Tocris; for mIPSCs) or 10 μM NBQX, 50 μM D-AP5 (for sIPSCs). Pipette solution contained (in mM): 135 CsCl, 10 Hepes, 0.2 EGTA, 2 ATP, 0.3 GTP, 10 Glucose (pH 7.3 with CsOH). For recording of sIPSCs 2 mM QX-314 (Tocris) was added to the pipette solution to inhibit spontaneous spiking of the recorded cell. Recordings and analysis used pClamp 10.4 software. See Supplementary Information for method of EPSC measurements.

### Head-fixed awake *in vivo* electrophysiology

The experimental procedure has been extensively described elsewhere^22^. In brief, mice were anaesthetised with isofluorane (3–4% induction, 1–2% maintenance). The skin overlaying the skull was removed under local anaesthesia using carbostesin (AstraZeneca, Zug, Switzerland). A steel head-post was then fixed on the bone by embedding its base in dental cement (Omni-Etch Dentin, OmniDent). The rest of the skull was also covered with dental cement except the part overlaying the OB. Animals were then put back to their cage and allowed to recover for a couple of days. Few days after recovery, mice were trained to be head-restrained for 2–4 sessions (30–60 min each) done in 2–3 days.

All odorants (ethyl valerate: EV, ethyl tiglate: ET, octanal: OA, hexanal: HX, L+, L−, octanol: OO and heptanol: HP) were obtained from Sigma-Aldrich. 4 ml of pure odorant or mixtures of odorants were placed in glass vials. Binary mixtures were made by mixing the odorant in liquid phase. Odorants were delivered for 1.5 s through a custom made olfactometer as described previously^22^,^43^,^71^. The odorant onset was set at the end of an inspiration. Airflow passed through the vials containing the odorants and was further diluted 20 times with clean dry air before being sent to the nose.

On the day of the experiment, mice were head restrained and a silicon-based recording electrode (A-4x2-Tet-5mm-150-200-312, NeuroNexus Technologies, Ann Arbor, MI, USA) was inserted as previously reported^22^. For all experiments, respiration was monitored using a bidirectional airflow sensor (AWM2100V, Honeywell, MN) placed in front of the mouse nose.

All subsequent analyses and statistics were done using custom routines written for Matlab (MathWorks, Inc., Natick, MA). For details see Supplementary Information.

### Olfactometry

A computer-controlled eight-channel liquid-dilution olfactometer (Knosys) was used for behavioural assessment of olfactory perception and discrimination abilities, as described previously^46^. For more details see Supplementary Information.

### Data analysis

Results are presented as means with s.e.m., unless stated otherwise. For statistical analyses normal distribution of results was tested using the Shapiro–Wilk test. Student's *t*-test or Mann–Whitney *U*-test was applied as appropriate. Results were considered statistically significant with *P* values <0.05. All experiments were performed blind with regard to the genotype of the mice.

### Data availability statement

The data that support the findings of this study are available from the corresponding authors on request.

## Additional information

**How to cite this article:** Gödde, K. *et al*. Disruption of Kcc2-dependent inhibition of olfactory bulb output neurons suggests its importance in odour discrimination. *Nat. Commun.* 7:12043 doi: 10.1038/ncomms12043 (2016).

## Supplementary Material

Supplementary Information Supplementary Figures 1-10 and Supplementary Methods
