# Supplementary material for: Disruption of Kcc2-dependent inhibition of olfactory bulb output neurons suggests its importance in odour discrimination
Source: Nat Commun. 2016 Jul 8;7:12043. doi: 10.1038/ncomms12043 (PMC4941119; doi:10.1038/ncomms12043)
Supplement: Supplementary Information — Supplementary Figures 1-10 and Supplementary Methods [file ncomms12043-s1.pdf]

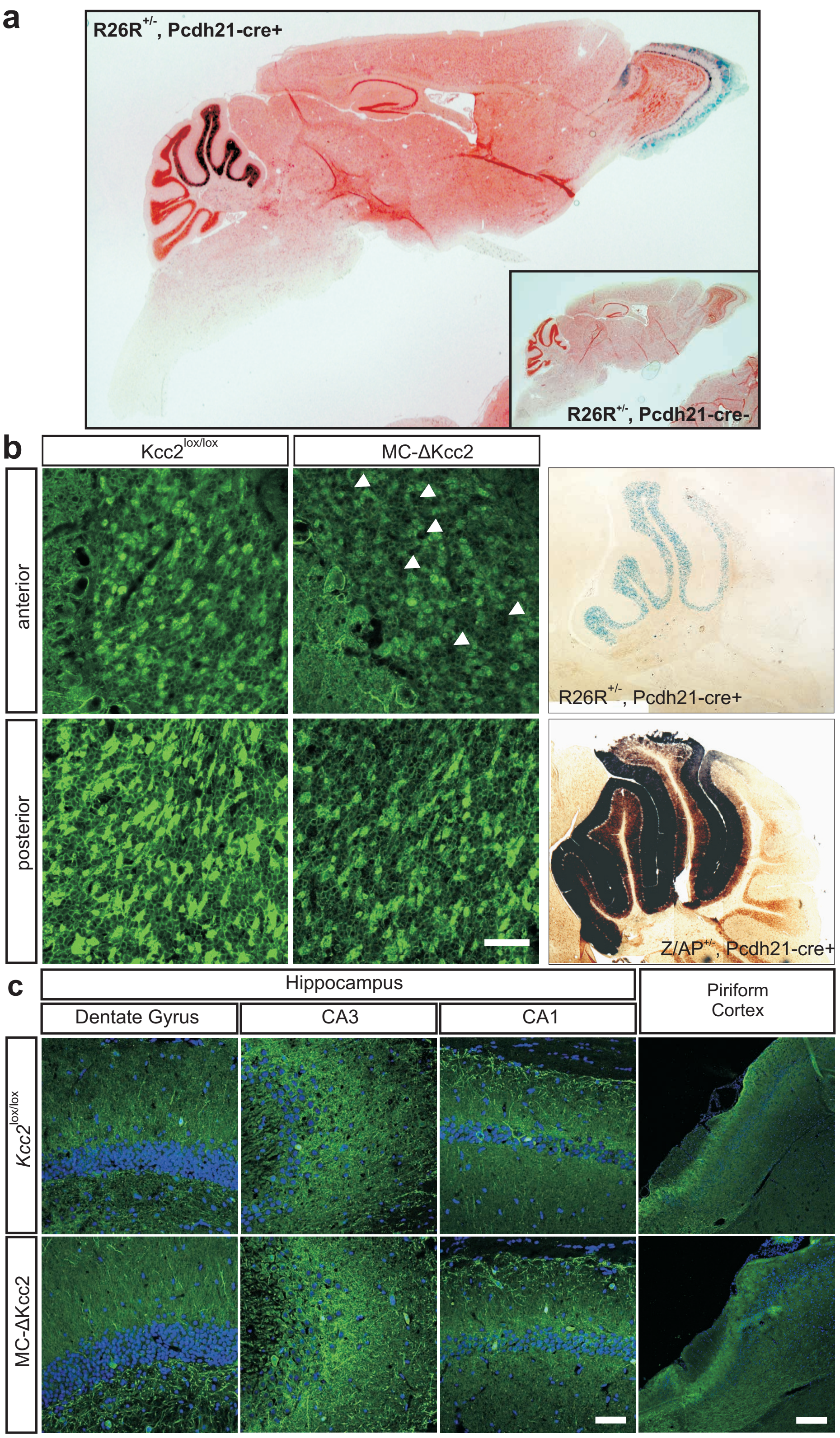

Supplementary figure 1 | Investigation of specificity of Pcdh21-cre line.

(a) X-Gal staining for ß-galactosidase acitivity in sagital slices of the brain from R26R reporter mice crossed to Pcdh21-cre mice shows staining of mitral and tufted cells in the OB, dorsal parts of the granular layer of the cerebellum and single cells in the cerebellar nucleus. Slices were counterstained with Neutral Red. Inset shows X-Gal staining in slices from R26R mice negative for Pcdh21-cre. (b) Immunofluorescent stainings of Kcc2 (green) in most anterior and posterior regions of the cerebellum in Kcc2$^{lox/lox}$ compared to MC-ΔKcc2 mice and reporter stainings of the cerebellum from Pdch21-cre mice crossed with R26R- and Z/AP reporter mice. Arrows indicate areas without obvious Kcc2 membrane staining,Scale represents 40 µm. (c) Immunofluorescent stainings of Kcc2 (green) in different regions of the hippocampus and of the piriform cortex in adult Kcc2$^{lox/lox}$ compared to MC-ΔKcc2 mice. Scale represents 50 µm (hippocampus) and 200 µm (piriform cortex).

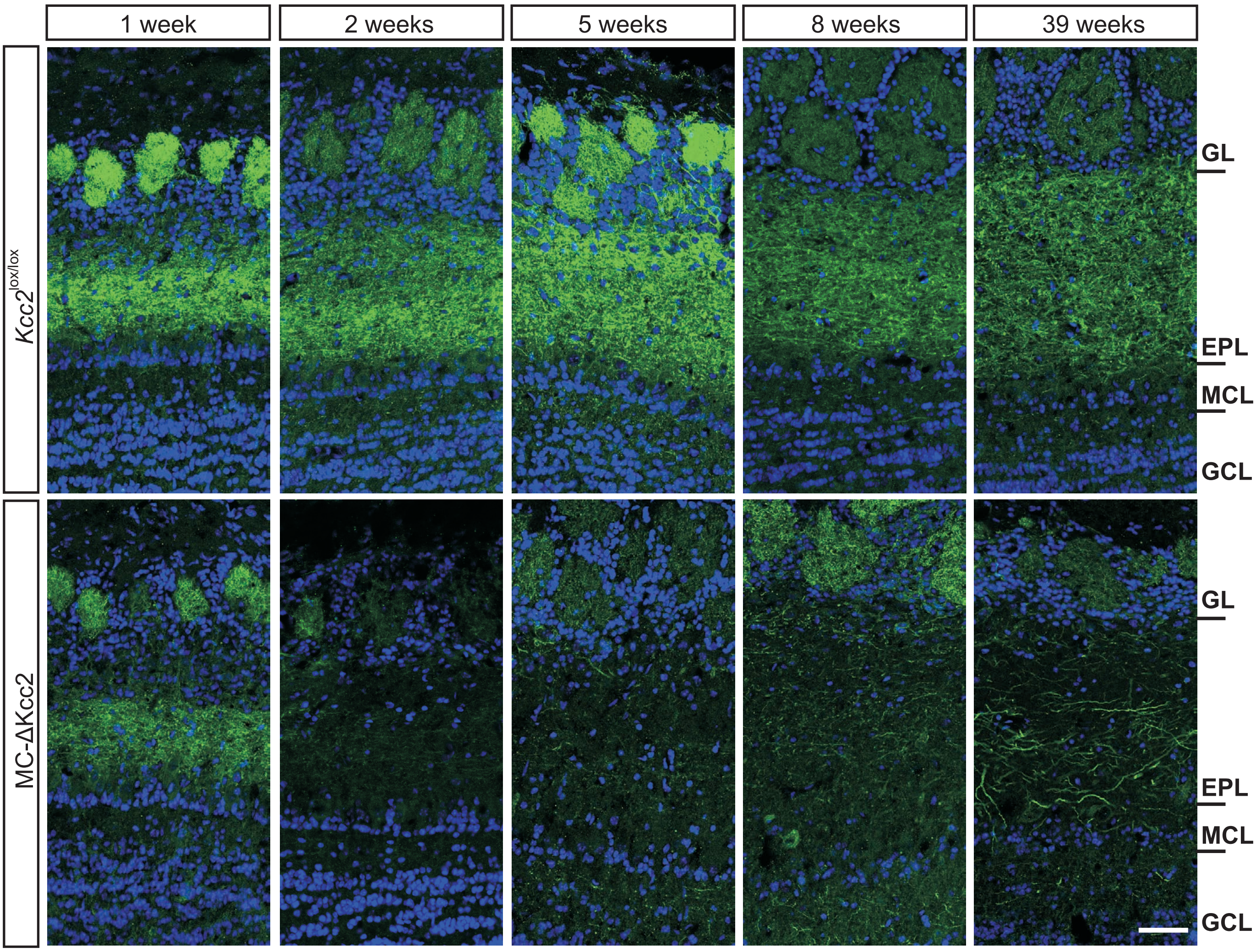

Supplementary figure 2 | Kcc2 deletion from mitral cells of MC-ΔKcc2 mice compared to *Kcc2*$^{lox/lox}$ mice at different ages.

Immunofluorescent labeling of Kcc2 (green) in 10 µm slices of olfactory bulbs of mice at ages of 1, 2, 5, 8 and 39 weeks. Nuclei were stained with DAPI (blue). Scale bar, 50 µm. GL, glomerular layer; EPL, external plexiform layer; MCL, mitral cell layer; GCL; granule cell layer.

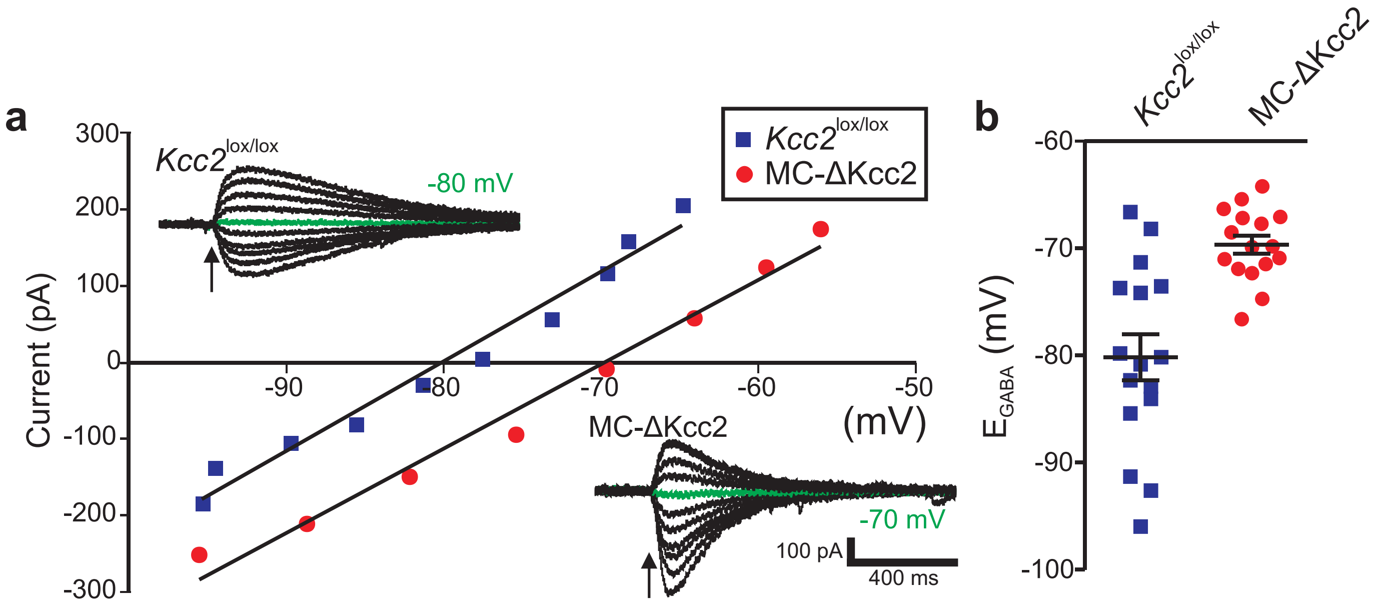

Supplementary figure 3 | Effect of *Kcc2* disruption on E_{GABA}.
(a) Muscimol-induced currents of mitral cells held at different voltages (from -130 mV to -40 mV in 10 mV steps for 10 sec) in perforated-patch clamp experiments. Maximal currents of typical cells induced by acute muscimol application plotted against voltage (after offline correction for access resistance). Intersection of the fitted line with I=0 indicates E_{GABA}. Original traces (baseline corrected) for either genotype shown as insets, with currents close to the reversal potential shown in green. Arrows indicate muscimol application. (b) Summary of E_{GABA} of MCs from MC-ΔKcc2 mice (n=16 cells) compared to *Kcc2*$^{lox/lox}$ control mice (n=16 cells) measured as in (a). Horizontal bars indicate mean ± SEM. Means are statistically different with a p-value < 0.0001, unpaired t-test.

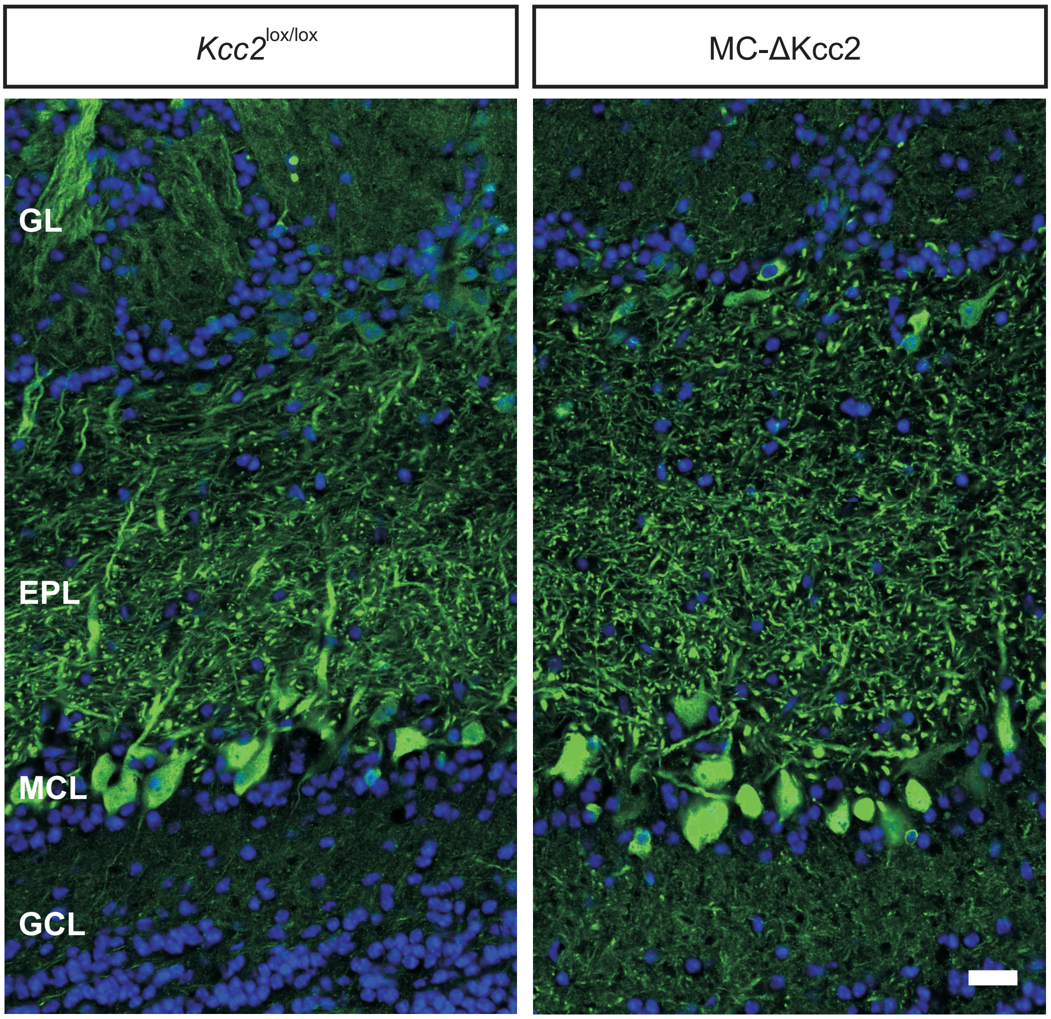

Supplementary figure 4 | MCs show no changes in gross morphology. Immunfluorescent labeling of the MC marker protein PGP9.5 (green) in slices (10µm) of the OB from MC-ΔKcc2 and *Kcc2*$^{lox/lox}$ mice. Nuclei are stained with DAPI (blue). Scale bar, 20 µm. GL, glomerular layer; EPL, external plexiform layer; MCL, mitral cell layer; GCL, granule cell layer.

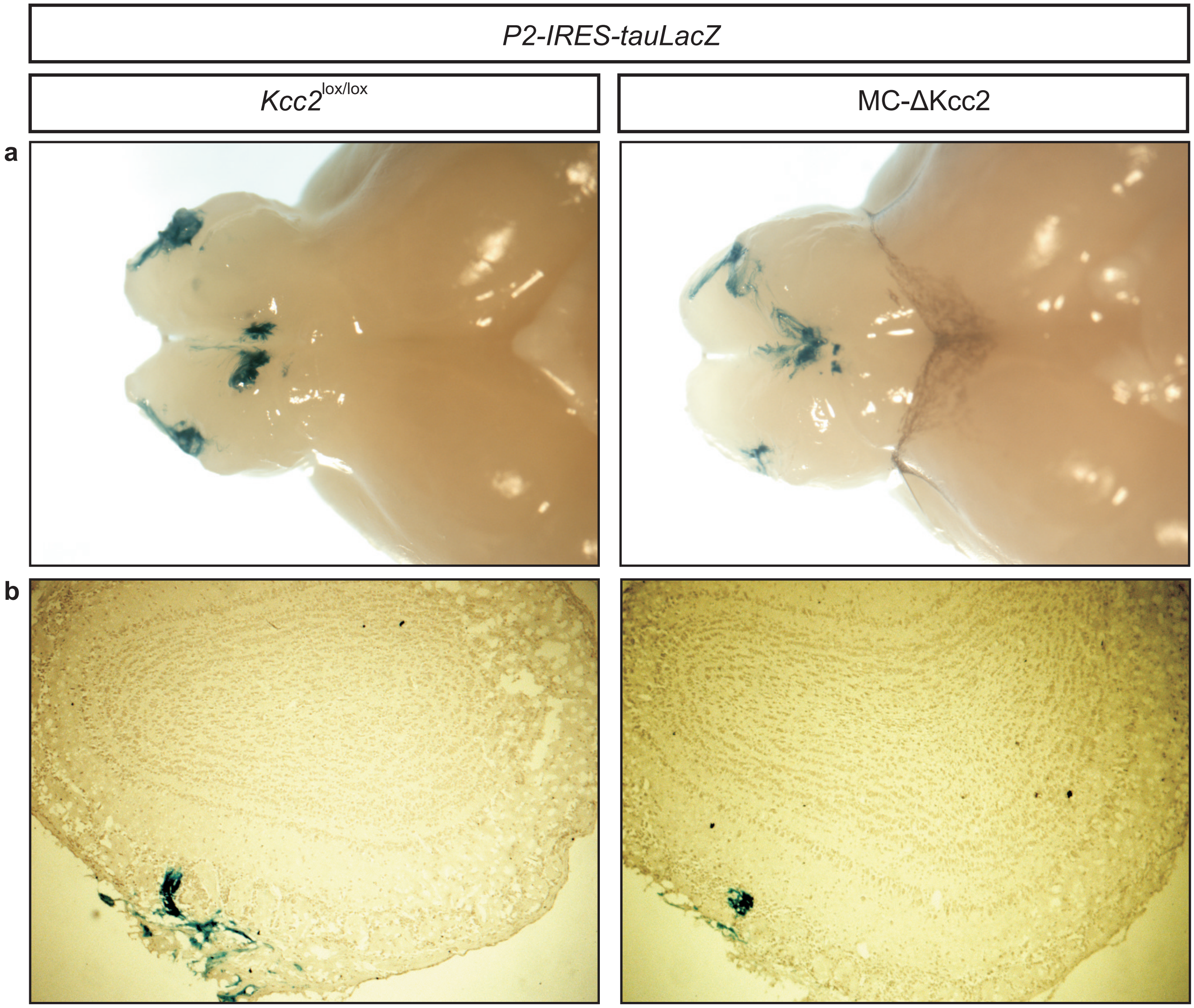

Supplementary figure 5 | Unchanged axonal coalescence in the OB of MC-ΔKcc2 mice. Axonal coalescence was investigated by X-Gal staining of the OB in MC-ΔKcc2 und *Kcc2*$^{lox/lox}$ mice crossed with P2-IRES-tauLacZ mice that express β-galactosidase under the control of the promotor for the olfactory receptor P2. (a) Both genotypes show axonal coalescence of P2-OSNs to 2 glomeruli ipsilateral in the OB, which are bilaterally symmetrically arranged. (b)Axonal coalescence detected in slices of the OB is comparable between genotypes.

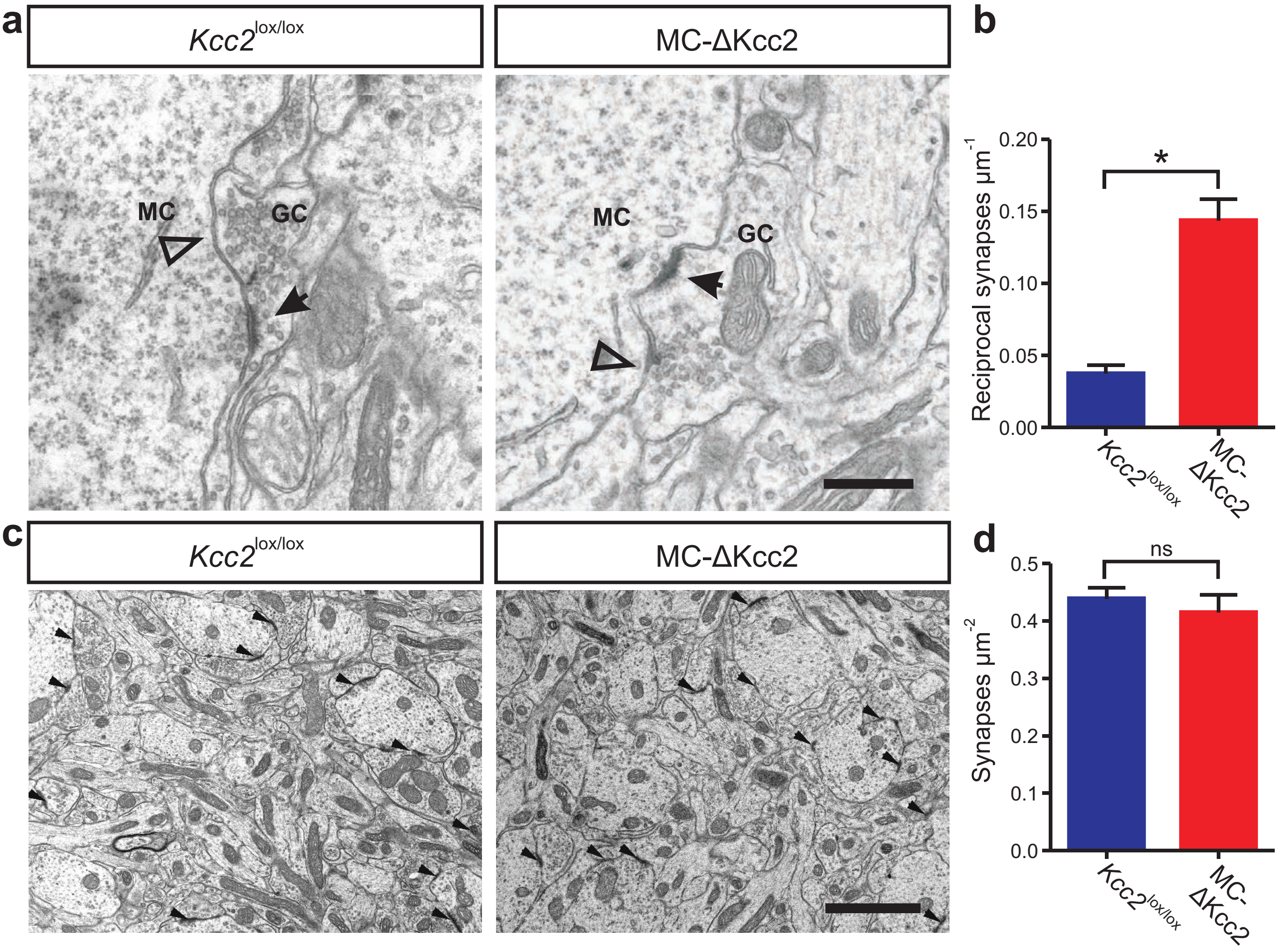

Supplementary figure 6 | Increased number of reciprocal synapses contacting the somata of mitral cells from MC-ΔKcc2 mice.

(a) Example electron micrographs of reciprocal synapses between mitral cells (MC) and granule cells (GC) in MC-ΔKcc2 mice and *Kcc2*$^{lox/lox}$ mice. Inhibitory components of the synapse with vesicles in the granule cell boutons are marked with open arrows. Excitatory component with vesicles clustered at the MC membrane and a pronounced synaptic density are marked with filled arrows. Scale bar, 500 nm (b) Average number of reciprocal synapses per membrane perimeter of MC-ΔKcc2 mice and *Kcc2*$^{lox/lox}$ mice. Reciprocal synapses were characterized by the abundance of opposing clustered vesicles at the side of the mitral cell as well as within the granule cell bouton. 4 mice per genotype and 8-10 cells per mouse were analysed. Mean + SEM is displayed. Means are statistically different with a p-value of 0.0286, Mann-Whitney test. (c) Example electron micrographs of the EPL neuropil from MC-ΔKcc2 mice and *Kcc2*$^{lox/lox}$ mice. Arrows indicate pre-synaptic boutons/post-synaptic density contacts. Scale bar, 2 µm (d) Average number of synapses per µm^2 in the EPL of MC-ΔKcc2 and *Kcc2*$^{lox/lox}$ mice. n=4 mice per genotype (in average 500 square microns of EPL neuropil area was analysed per animal) were analyzed. Mean + SEM are displayed.

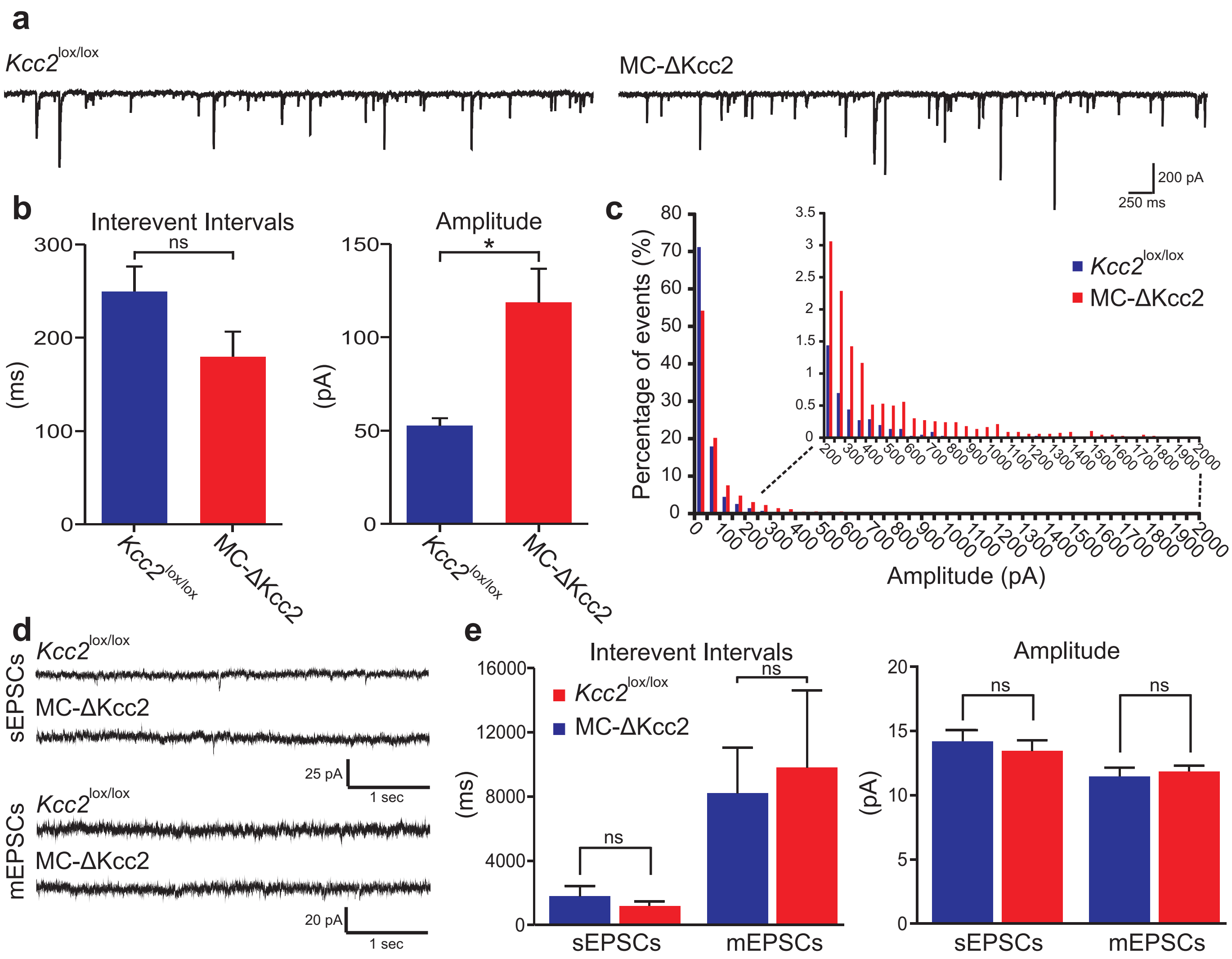

Supplementary figure 7 | Effect of Kcc2 deletion from mitral cells on spontaneous inhibitory post-synaptic currents and excitatory currents in mitral cells.

(a) Sample traces of sIPSCs measured in mitral cells of MC-ΔKcc2 mice and *Kcc2*$^{lox/lox}$ mice at a potential of -70 mV. (b) Mean Interevent Intervals of sIPSCs were not significantly changed in MC-ΔKcc2 mice (n=26 cells) compared to *Kcc2*$^{lox/lox}$ mice (n=29 cells). Mitral cells of MC-ΔKcc2 had significantly increased amplitudes of sIPSCs compared to *Kcc2*$^{lox/lox}$. Data is shown as mean ± SEM. Means were statistically different with a p-value < 0.0001, Mann-Whitney Test. (c) Frequency histogram of sIPSC amplitudes. Inset, histogram with different scale for amplitudes >200 pA. Data was calculated from 6596 events for either genotype with a bin size of 50 pA. Distributions differ with an asymptotic significance (2-sided) < 0.0005 (Kolmogrorov-Smirnov test).(d) Sample traces of sEPSCs and mEPSCs measured in mitral cells of MC-ΔKcc2 mice and *Kcc2*$^{lox/lox}$ mice at a potential of -75 mV. (e) Mean Interevent Intervals and amplitudes of sEPSCs and mEPSCs were not significantly changed in MC-ΔKcc2 mice (n=8 cells for sEPSCs and 6 cells for mEPSCs) compared to *Kcc2*$^{lox/lox}$ mice (n=10 cells for sEPSCs and 8 cells for mEPSCs). Data is shown as mean ± SEM.

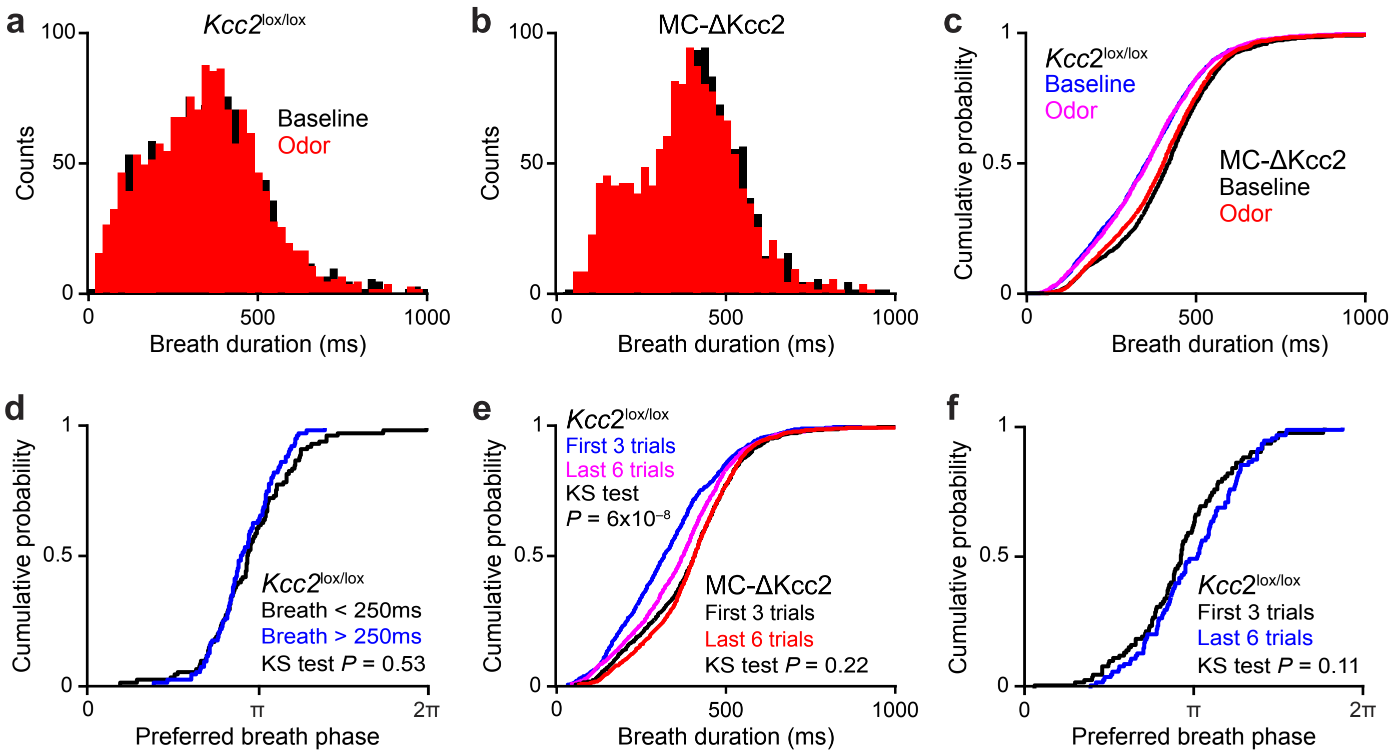

Supplementary figure 8 | Breathing patterns in Kcc2$^{lox/lox}$ and MC-ΔKcc2

(a-b) Distributions of breath duration during baseline and odorant application in Kcc2lox/lox and MC-ΔKcc2 mice (all cell-odor pairs pooled). (c) Cumulative probability distributions of breath duration during baseline and during odorant application in Kcc2$^{lox/lox}$ and MC-ΔKcc2 mice. For each genotype, baseline and odor distributions are not different (Kolmogorov-Smirnov test P = 0.94 and 0.053 for Kcc2$^{lox/lox}$ and MC-ΔKcc2 respectively). MC-ΔKcc2 mice are doing significantly less fast sniffing (Kolmogorov-Smirnov test P = 7x10−11). (d) Cumulative probability distributions of preferred phases of firing computed for M/T cells recorded in Kcc2$^{lox/lox}$ during odorant period either for breaths of either short (<250ms) or long durations (>250ms). No significant difference between the distributions. (e) Cumulative probability distributions of breath duration during odorant period for the first three and the last six trials of the same odorant in Kcc2$^{lox/lox}$ and MC-ΔKcc2 mice. (f) Cumulative probability distributions of M/T preferred phases of firing for the first three and the last six trials of the same odorant in Kcc2$^{lox/lox}$.

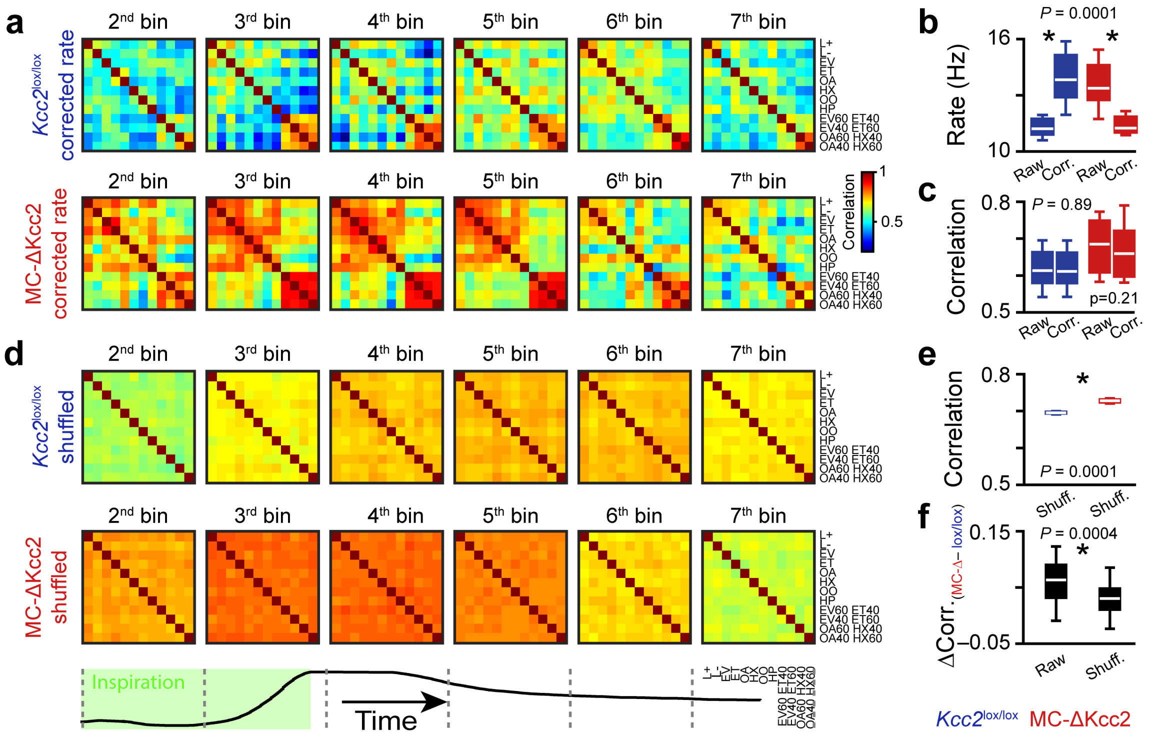

Supplementary figure 9 | Differences of ensemble correlation between Kcc2$^{lox/lox}$ and MC-ΔKcc2 mice cannot be explained by differences in absolute firing rate

(a) Temporal evolution of the correlations for all possible pairs of mixtures during the 1st breath after odor onset for rate-corrected data. The firing rate of Kcc2$^{lox/lox}$ and MC-ΔKcc2 cells was corrected to compensate for the absolute rate difference by adding or subtracting the delta rate (plus some variance) observed between KO and WT populations, respectively. (b) Bar graph showing the firing rate evoked by odorants for the raw and corrected data (Wilcoxon paired test). The corrected Kcc2$^{lox/lox}$ cell rate was significantly increased compared to its raw rate (Wilcoxon paired test P = 0.0001) and thereby resembled the raw rate of MC-ΔKcc2 cells (Mann-Whitney test P = 0.072) We observed the exactly opposite tendency for MC-ΔKcc2 cells (Wilcoxon paired test P = 0.0001, Mann-Whitney test P = 0.081) (c) Bar graph showing the average correlation computed over the first breath after odor onset for all odorant pairs for the raw and corrected data. Strikingly, this correction maintained the temporal structure of the firing patterns and consequently led to a similar pattern correlation for both genotypes (Wilcoxon paired test, Kcc2$^{lox/lox}$, P = 0.89 and MC-ΔKcc2, P = 0.21) (d) Temporal evolution of the correlations for all possible pairs of mixtures during the 1st breath after odor onset for the raw data which have been shuffled (odorant information was randomly shuffled for each cell). This operation completely abolished the correlation patterns observed in normal conditions (e) Bar graph showing the average correlation computed over the first breath after odor onset for all odorant pairs for the shuffled data (Mann-Whitney test). Shuffling significantly increased the correlation in MC-ΔKcc2 mice (Mann-Whitney test P = 0.0001) (f) Difference of correlation computed between Kcc2$^{lox/lox}$ and MC-ΔKcc2 data for the raw and shuffled data (Wilcoxon paired test). The difference was significantly smaller than the one observed with the raw data (Wilcoxon paired test, P = 0.0004). In summary, the differences of rate alone cannot explain the differences of correlation observed between the two genotypes.

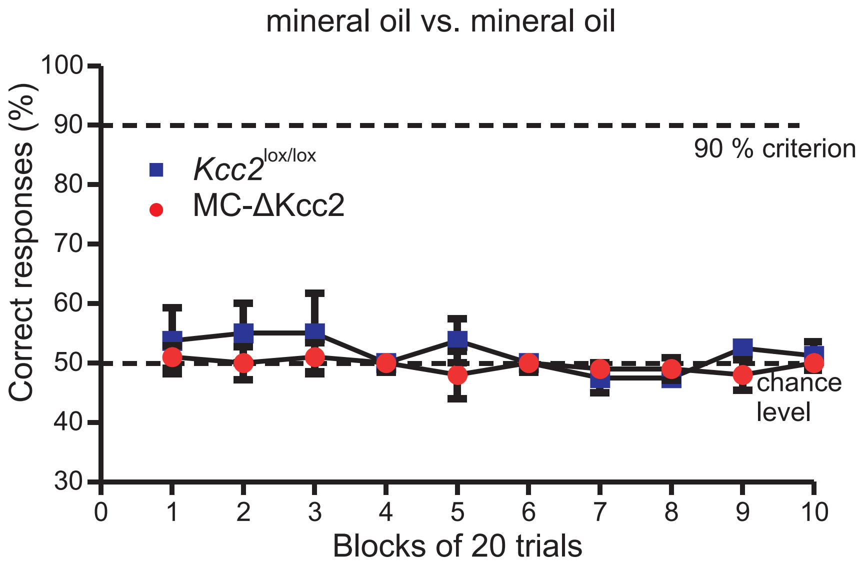

Supplementary figure 10 | Control measurement for odor discrimination tests in the olfactometer.

Mice of each genotype (MC-ΔKcc2: n=5 mice, *Kcc2*$^{lox/lox}$: n=4 mice) did not perform better than chance level when tested for mineral oil vs. mineral oil. This excludes a bias of the olfactometry results by external cues.

Supplemental methods

Determination of OSN coalescence

P2-IRES-tauLacZ (Mombaerts et al., 1996) mice were crossed to *Kcc2*$^{lox/lox}$ and Pcdh21::Cre mice. Experiments were carried out with 5 weeks old mice. The mice were perfused with PBS followed by 4% PFA (w/v) in PBS. Brains were post-fixed (20 min on ice), washed in buffer A (100 mM phosphate buffer (pH 7.4), 2 mM $MgCl_2$, 2.5 mM EGTA) and incubated for 30 min in buffer A with 0.02% NP-40 (v/v) and 0.01% deoxycholate (w/v). LacZ staining was carried out overnight in buffer A with 0.2% NP-40 (v/v), 0.1% deoxycholate (w/v), 5 mM potassium ferricyanide 5 mM potassium ferrocyanide and 1 mg/ml of X-Gal at room temperature (20–25°C). Whole-mount stained bulbs were cryoprotected in 30% sucrose (w/v), frozen in Tissue-Tek O.C.T. compound (Sakura) and cut into 20-µm cryostat sections. Sections were post-stained with X-Gal and counterstained with Neutral Red.

X-Gal staining for LacZ activity

X-Gal staining for ß-galactosidase activity was performed on 10 µm sagittal slices of the brain. Slices were incubated in LacZ buffer (2 mM $MgCl_2$, 5 mM EGTA, 0.02% NP-40, 0.01% Na-deoxycholate in 0.1 M phosphate buffer) for 2x 5 min. X-Gal staining solution (2 mM $MgCl_2$, 5 mM $K_3Fe(CN)_6$, 5 mM $K_4Fe(CN)_6$, 0.2% NP-40, 0.1%Na-deoxycholate in 0.1 M phosphate buffer) was added and slices were incubated in the dark for 5-18 h at 37°C. Staining reaction was stopped by washing the slides in PBS. A neutral red counterstaining was performed using 0.5% Neutral Red solution in acetate buffer (Sigma Aldrich).

Alkaline phosphatase staining in Z/AP report mice

For alkaline phosphatase staining slices were incubated for 1 h at 60°C for heat inactivation of endogenous phosphatases. After washing with AP buffer (100 mM NaCl, 100 mM Tris/Cl pH9.5, 50 mM MgCl2, 1% Tween-20, 2 mM Levamisole) slices were incubated with NBT/BCIP solution until appropriate staining intensity was reached.

Measuring EPSCs in acute slices of the OB

sEPSCs and mEPSCs were measured in the whole cell configuration. MCs were clamped at -75 mV. For measurements of mEPSCs ACSF was supplemented with 1 μm TTX. Pipette solution contained (in mM): 130 Cs-methanesulfonate, 7 NaCl, 2 Mg-ATP, 10 Hepes, 2 QX-314. We used pClamp 10.4 software for recordings and analysis.

***In vivo* electrophysiological data analysis**

Statistical analysis of the rate change for single cell responses

The analysis procedure has been extensively described elsewhere[22]. In brief, change of odor-evoked firing rate over the breathing cycle duration was assessed, relative to baseline, by the non-parametric Wilcoxon rank sum (WRS) test repeated in each respiratory cycles spanning stimulus presentation and for all cells and stimuli. Three breathing cycles in the baseline activity were used as a template. The template was compared subsequently to the first three breathing cycles after odor onset. In a particular cycle, a cell was considered as responsive if at least one odorant stimulus evoked a significant change in firing in comparison to baseline. We set the p-value to 0.05.

For each significant change, a response was considered as excited if the average firing rate over the complete breathing cycle was significantly higher from the baseline and inhibited if it was significantly lower.

Statistical analysis of the temporal change for single cell responses

The analysis procedure has been extensively described elsewhere[22]. In brief, change in the spike timing distribution during odor presentation was assessed by comparing the spike timing relative to the onset of the inspiration before and after odorant presentation using a Kolmogorov-Smirnov (KS) test repeated for each respiratory cycles for all cells and stimuli. Three breathing cycles in the baseline activity were used as a template. The template was compared each consecutive breathing cycle (3 in total) after the odor onset. In a particular cycle, a cell was considered as responsive if at least one odorant

stimulus evoked a significant change in firing in comparison to baseline. Results were considered statistically significant with a p-value < 0.05.

Statistical analysis of MC sniff-phased activity

Spike timings were transformed into corresponding radian values related to the breath cycle. The preferred phase and its significance were calculated using a circular Raleigh test for non-uniformity. Only the cells, which had a significant phase of spiking were taken into consideration. To test the significant change of phased activity between the baseline and the odor epoch, we used a circular Watson Wheeler test. Only cells which had a significant change of phase were considered. For both tests, spikes from one breath pre-odor and the first breath post-odor were considered for the calculation of the preferred phase in the baseline and the odor period. Results were considered statistically significant with a P-value < 0.05.

Population vector construction

We pooled all M/T cells recorded in different animals. The activity of the 89 and 121 neurons measured in *Kcc2*$^{lox/lox}$ and MC-ΔKcc2 mice mice, respectively, were organized in 89 and 121 dimensional vectors respectively, containing in each dimension the average firing rate of a recorded cell computed over a certain time bin. Population vectors were built using 1 and 8 bins per breathing cycles (on average for the 8 bins, 40 and 47 ms per bin in *Kcc2*$^{lox/lox}$ and MC-ΔKcc2 mice mice, respectively).

Correlation matrix

The analysis procedure has been extensively described elsewhere[22]. In brief, the matrix of correlation was built by computing the Pearson coefficient of correlation between pairs of population vectors averaged over the trials evoked by two different odors. The dimensions of the matrix correspond to n x n, where n is the number of odors. Each square corresponds to the Pearson coefficient of correlation of a particular odor against another. The odorants were ranked based on the theoretical ratios of components.

Prediction algorithm

To compute classification performance, one trial per stimulus was chosen to be a test set, and the remaining trials were averaged to be template responses. The Euclidean distances between test trials and all stimuli templates were computed, and trials were assigned to the closest template (i.e. to a stimulus prediction). The percentage of success for odor identity and intensity was the fraction of correct assignments over the total number of assignments. We averaged this percentage over all odors used.

In short, the algorithm creates template vectors for each stimulus based on a fraction of the stimulus repetitions and then assigns each remaining trial to the closest template (i.e. measuring the Euclidean distance).

Random rate correction

Firing patterns of WT cells were artificially corrected in order to mimic those of KO cells, while preserving their temporal structure (e.g. preferred phase), and vice-versa. For each WT or KO cell, we respectively added or subtracted the delta rate (plus or minus its variance) between KO and WT populations. More precisely, for each trial (tr) of any given odorant (od) and at each time bin (t) of the first breath, we computed the mean population firing rate for both WT ($R_{t,od\ WT}$) and KO ($R_{t,od\ KO}$). We then calculated the mean delta rate (i.e. mean $\Delta R_{tr} = R_{t,od,KO} - R_{t,od\ WT}$) and standard deviation (SD ΔR_{tr}) across all trials. Based on the mean and SD values, we created over the entire cell population an artificial distribution of rate (following either a normal or a Poisson distribution which gave similar results) that we either added or subtracted to the rate of WT and KO cells respectively. This operation was randomly repeated for each trial of a given odorant. These corrected rates were then used to compute the correlation matrices.

In order to test a possible contribution of the rate to the higher levels of correlation in the KO population, we shuffled for each cell the identity of the odor evoked response and recreated a population vector for further analyses. This process has been randomly repeated 20 times and the results averaged.

Olfactometry

After training of mice with the BEGIN program, we used the D2 program with standard settings for testing. Chemicals were from Sigma-Aldrich/Fluka. Mice (8-35 weeks old, male or female) were tested on the following tasks: discrimination of structurally different odorants (1% ethyl valerate vs. 1% ethyl tiglate; 1% octanal vs. 1% hexanal), discrimination of structurally similar odorants (1% (-)-limonene vs. 1% (+)-limonene; 1% octanol vs. 1% heptanol) and discrimination of mixtures of the same odors (0.6/0.4% ethyl valerate/ethyl tiglate vs. 0.4/0.6% ethyl valerate/ethyl tiglate; 0.6/0.4% octanal/hexanal vs. 0.4/0.6% octanal/hexanal). All odorant were diluted in mineral oil. As a negative control we tested mineral oil vs. mineral oil. Each test run includes 10 trials of 20 odor presentation. Percentage of correct answers per trail was displayed.
